# A BAC pooling strategy combined with PCR-based screenings in a large, highly repetitive genome enables integration of the maize genetic and physical maps

**DOI:** 10.1186/1471-2164-8-47

**Published:** 2007-02-09

**Authors:** Young-Sun Yim, Patricia Moak, Hector Sanchez-Villeda, Theresa A Musket, Pamela Close, Patricia E Klein, John E Mullet, Michael D McMullen, Zheiwei Fang, Mary L Schaeffer, Jack M Gardiner, Edward H Coe, Georgia L Davis

**Affiliations:** 1 Division of Plant Sciences, University of Missouri, 1-31 Agriculture, Columbia, MO, USA; 2 Institute for Plant Genomics and Biotechnology, Texas A&M University, College Station, TX, USA; 3 USDA-ARS, PGRU, 210 Curtis Hall, Columbia, MO, USA; 4 Department of Plant Sciences, 303 Forbes Hall, University of Arizona, Tucson, AZ, USA

## Abstract

**Background:**

Molecular markers serve three important functions in physical map assembly. First, they provide anchor points to genetic maps facilitating functional genomic studies. Second, they reduce the overlap required for BAC contig assembly from 80 to 50 percent. Finally, they validate assemblies based solely on BAC fingerprints. We employed a six-dimensional BAC pooling strategy in combination with a high-throughput PCR-based screening method to anchor the maize genetic and physical maps.

**Results:**

A total of 110,592 maize BAC clones (~ 6x haploid genome equivalents) were pooled into six different matrices, each containing 48 pools of BAC DNA. The quality of the BAC DNA pools and their utility for identifying BACs containing target genomic sequences was tested using 254 PCR-based STS markers. Five types of PCR-based STS markers were screened to assess potential uses for the BAC pools. An average of 4.68 BAC clones were identified per marker analyzed. These results were integrated with BAC fingerprint data generated by the Arizona Genomics Institute (AGI) and the Arizona Genomics Computational Laboratory (AGCoL) to assemble the BAC contigs using the FingerPrinted Contigs (FPC) software and contribute to the construction and anchoring of the physical map. A total of 234 markers (92.5%) anchored BAC contigs to their genetic map positions. The results can be viewed on the integrated map of maize [1,2].

**Conclusion:**

This BAC pooling strategy is a rapid, cost effective method for genome assembly and anchoring. The requirement for six replicate positive amplifications makes this a robust method for use in large genomes with high amounts of repetitive DNA such as maize. This strategy can be used to physically map duplicate loci, provide order information for loci in a small genetic interval or with no genetic recombination, and loci with conflicting hybridization-based information.

## Background

Maize (*Zea mays*) is a member of the Gramineae family. It has a relatively large genome size of 2,500 Mb, which is comparable to the human genome [3], but is larger than some other grass plants, 430 Mb for rice and 750 Mb for sorghum [4]. The genome sizes of many plant species differ according to the amount of repetitive DNA [5]. In maize repetitive sequences are estimated to comprise well over 50% of the genome [5-7]. Maize is a challenging target for genome analysis because of its large genome size, duplication of genomic regions [8], low percentage of single-copy DNA, and high retro-element content [6].

Integrated genetic and physical maps are extremely valuable for positional cloning of agriculturally important genes, comparative genome analysis, studies of chromosomal organization, and clone-by-clone sequencing. Various methods have been developed for integrating genetic and physical maps of complex genomes. Hybridization-based screening can be performed using high-density filters of large insert clones and radioactively labeled cloned DNA fragments, PCR-amplified products, or DNA oligonucleotides. More recently, overgo hybridization probes resulting from annealing of two overlapping oligonucleotides (followed by a fill in reaction) have been employed to generate BAC contig maps [9,10]. This technique has an advantage over the conventional oligonucleotide probe hybridization method, because the probes are slightly larger, providing improved hybridization kinetics and specificity. However, hybridization-based screening approaches have several limitations over PCR-based screening methods. The presence of repeat elements in the labeled probe often confounds hybridization results, [11] and the procedures are more cumbersome since they involve radioactive material.

PCR-based screening of large insert libraries based on STS markers provides an alternative to hybridization-based screens. The efficiency of PCR-based screening can be improved by pooling clone libraries in specific ways. Once pooled, clones containing particular sequences can be located by identifying the subset of pools containing the corresponding STS markers [12-14].

BAC pools are also an alternative to the use of radiation hybrids since both approaches obviate the need for DNA polymorphism in mapping loci to their chromosomal locations at high-resolution. However, the BAC pool and radiation hybrid approaches have some differences. For example, individual chromosome segments in BAC pools tend to be smaller, giving finer map resolution, and individual BAC pools tend to contain a larger number of unlinked chromosome segments than individual radiation hybrids, which increases the likelihood of false positive associations [15]. The increased rate of false positives can be overcome by using pools with six dimensions instead of the conventional two or three dimensions [14].

In this study, we describe an approach to integrate the maize physical and genetic maps by using PCR-based STS markers generated from maize sequences to screen BAC DNA pools. The resulting data were analyzed together with BAC fingerprint data in FPC for contig assembly [2]. The integrated map arising from this data is displayed in iMap [1].

## Results

### Pooling strategy

**Figure 1:**
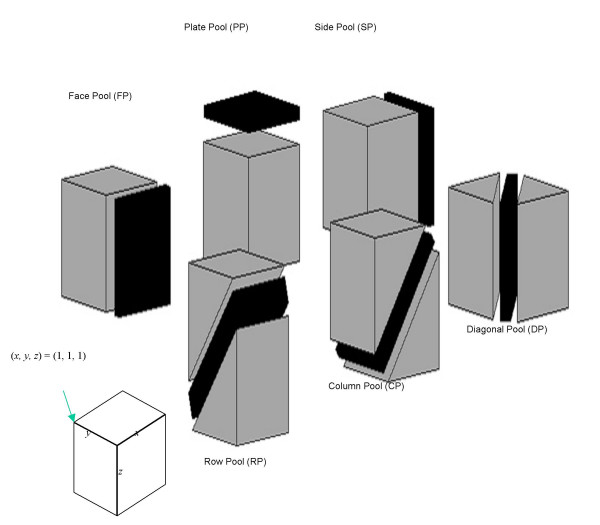
**Schematic display of BAC pooling strategy for six different matrices**. Two hundred eighty eight 384-well microtiter plates containing 110,592 individual BAC clones were arranged in a three-dimensional square consisting of 48 ranks in all three axes (*x*, *y*, and *z*). Each pooled BAC DNA consists of DNAs isolated from 2,304 BAC clones simultaneously.

The pooling design is illustrated in Fig. 1. The pooling method used was modified from the one used in sorghum [14] to accommodate the larger genome size of maize (about 3× of sorghum). A total of 110,592 BAC clones were pooled in six distinct directions to generate 288 unique pools (48 per dimension) containing 2,304 clones (one-eighth of a genome equivalent).

### Contig assembly and anchoring via high-throughput PCR screening of pools

**Figure 2:**
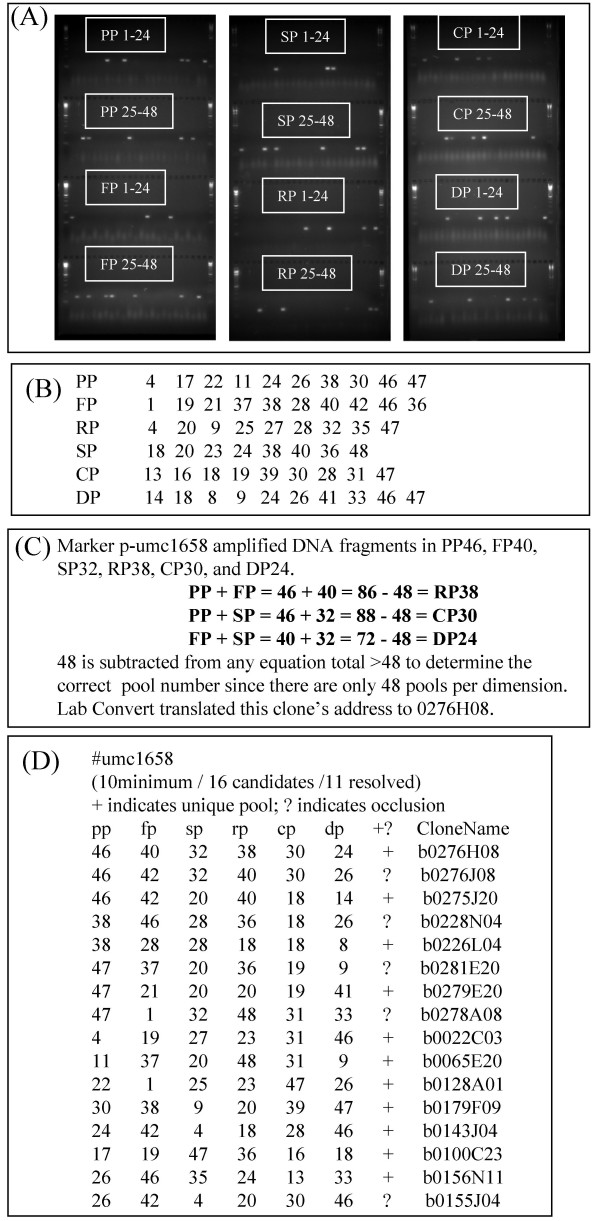
**Representative gel images of BAC pool screening**. Primers for umc1658 were used to amplify pool DNA. On average 6 positives per dimension are expected. Amplified PCR products were electrophoresed on 4.5% Super Fine Resolution Agarose gels (A). Each gel contained two dimensions of BAC pools, first gel with plate (PP) and face (FP) pools, second gel with side (SP) and row (RP) pools, and the last gel on the right with column (CP) and diagonal (DP) pools. First and last lane of each tier contains 100 bp ladder. Electrophoresed gel images were scored in tab delimited text format (B). Amplified products were deconvoluted by Resolve script using 3 equations (C). BAC addresses were automatically converted using Lab Convert software (D).

**Figure 3:**
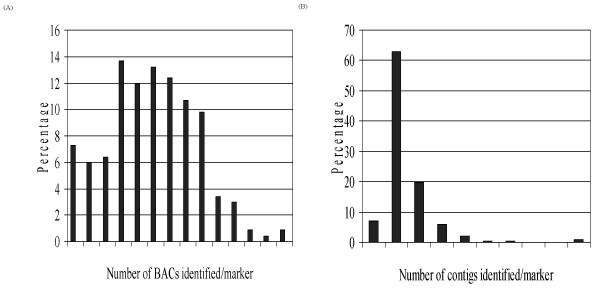
**Distribution of BAC, Contig/marker hits**. Maize *Hin*dIII BAC pools (6×) were screened with 237 PCR-base STS markers. The distribution of BAC hits/marker using all 237 markers (A) is illustrated. Among 237 markers tested, marker and BAC association data from 197 markers, and map position on the IBM genetic map, were used for the analysis of distribution of the numbers of identified contigs for each marker (B).

DNA pools were screened with 254 PCR-based STS markers to identify overlapping BAC clones. All 254 primer pairs amplified products from total B73 genomic DNA (data not shown) that were assumed to represent a single locus in the maize genome for each primer pair. Six BAC clones on average are expected to contain the same locus in each dimension of the pools because each BAC pool is about six genome equivalents (a representative image is provided in Fig. 2). An average of 4.68 BAC clones were identified per STS marker screened on the BAC DNA pools (Fig. 3A). Seventeen out of 254 (6.7%) markers gave no amplification products from the BAC pools. This is surprising because a ~ 6× library should have over 99% representation of all genome sequences.

Data from the 237 markers with genetic map locations were used to analyze the number of contigs identified per marker (Fig. 3B) and to assess the anchoring of these contigs to the maize genetic map. Of 237 markers, 149 (63.4%) identified BACs within a single contig and 55 (23.4%) identified BACs within two contigs. Thirty markers (12.7%) were identified within three or more contigs. Three markers (1.3%) identified BACs not included in the contig assembly due to fingerprinting failure. Marker to BAC relationships are provided. [See additional file 2]. Using conservative anchoring rules [16], 191 markers (80.6%) anchored contigs to their genetic position on the IBM (Intermated B73 × Mo17) map.

Markers that identified multiple contigs provide candidates for merging BAC contigs and for distinguishing duplicated genomic regions. For example, p-umc2046 identified BACs assembled within two contigs prior to manual editing of the physical map (data not shown). These BAC were located at the edges of the two contigs. At the high stringency used for maize contig assembly in FPC (cutoff value of 1 × 10^-12^) [17], BAC clones with a minimal fragment overlap such as these will not be placed in the same contig without additional marker information. The two contigs associated with p-umc2046 were merged by manual editing based on agreement of pool data and *Hin*dIII fingerprinting data (contig #201).

### Other utilities of BAC pools

#### Monomorphic markers

Three different types of markers were used to investigate additional utilities for the BAC pools. Nineteen monomorphic SSRs were screened through the BAC pools to determine their relative positions on the maize genome map by comparison with other anchored markers and to test the feasibility of BAC pools as an alternative to radiation hybrid mapping.

All thirteen of the monomorphic SSR markers had marker:BAC associations, twelve hit contigs that contain other genetic markers and twelve markers hit contigs that contain telomere or knob repeats (data not shown). Three markers identified BACs present in anchored contigs which are mapped to bins 3.07 (~ 567.6 cM), 7.03 (between 387.5 to 390.5 cM) and 5.01 (~ 124.7 cM), respectively.

#### Markers with low recombination rates

SSR markers with a low recombination rate on the IBM genetic map were used to examine the resolution of the IBM genetic map. Eighteen pairs of markers from five chromosomal regions were selected because the recombination rate between them is < 10 % on the IBM genetic map (equivalent to ~; 3% on conventional mapping populations). Their sequences were retrieved from GenBank and used as queries in a BLAST search against all maize sequences to confirm that these linked SSR markers were not designed from the same gene. None of the markers represented the same gene or had significant hits to other adjacent SSRs. Table 1 provides a summary of the contigs identified. Five bins (1.05, 3.04, 5.06, 6.04, and 7.03) containing markers with low recombination rates where contigs that were hit by multiple adjacent genetic markers were examined. In bin 3.04 the following markers show a possible rearrangement in order from *bngl1019-(umc1717, bnlg1452, bnlg1113)-umc1655 *where the markers in parenthesis have the same map coordinate to *bnlg1113-umc1777-(bnlg1019, bnlg1452, umc1655) *where the markers in parentheses are on the same BAC clone. Data from markers in bin 5.06 also suggested an order rearrangement from *umc1941-umc1680-umc1542-phi087-umc2306-bnlg609 *to *umc1524-umc1680-umc1941-phi087-bnlg609-umc2306*. In addition, *umc1301 *and *umc1936 *map genetically on chromosome 7 at 405.5 cM. BAC pool data indicates that the correct marker order for these genes is *umc1936 *then *umc1301*.

#### Surveying transcription factors from gene families

PCR primers designed from 16 transcription factors were used to demonstrate how rapidly a gene family of interest could be mapped to BAC contigs and to evaluate the pools for studying duplicated regions of the genome (Table 2). Primers were designed to amplify locus-specific products. The transcription factors involved in the anthocyanin biosynthesis pathway have been extensively studied in maize [20-23] but little information is known about the genetic map positions of many other transcription factors, especially for duplicated genes. All 16 transcription factors identified multiple BAC clones within contigs (Table 2). Two pairs of paralogous genes, *C1/Pl *and *R1/B*, generated by the duplication that occurred during the allotetraploid origin of the maize genome [24], hit different contigs.

### Resolution of overgo hybridization ambiguities

**Figure 4:**
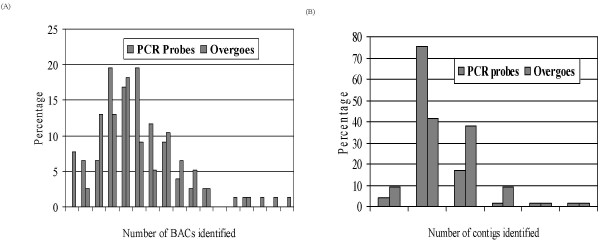
**Distribution of BAC, Contig/marker hits**. BAC and contig data from both the PCR-based screening on pool and overgo hybridizations are illustrated for the distribution of BAC hits/marker (A) and contig hits/marker (B) for 81 markers.

Sequences from which overgoes had been derived were analyzed against the UniGene database by BLAST analysis to identify Unigenes that contained an overgo and a genetically mapped SSR or RFLP. A total of 81 overgo probes with *in silico *connections to mapped SSR markers that could not displayed on iMap (because of conflicts or lack of positives) were selected for this study. SSR markers associated with these overgo probes by *in silico *connections were screened through the DNA pools to resolve overgo hybridization ambiguities and *in silico *associations (Fig. 4A and 4B). All 81 PCR-based markers identified at least one BAC clone, compared to six (7.4%) overgos with no positive BAC. On average, PCR screening of BAC pools and overgo hybridization identified 4.7 and 5.3 BAC clones per probe analyzed, respectively. Despite the fact that the overgo hybridizations were performed on a smaller subset of the *Hin*dIII BAC library (4.5× genome equivalents) compared to PCR screening on BAC pools (6× genome equivalents), the overgo hybridization identified significantly more BACs on average. This may result from repeat elements in the overgo probe hybridizing to multiple loci or a higher rate of false negatives in the pools compared to the overgo method. False-negatives were found in high frequencies in the pool dataset when the marker was present in ten or more pools of a given dimension (data not shown). During deconvolution, these clones were occluded because they shared *x, y, and z *coordinates with at least one other candidate BAC clone. Seventy-one out of 81 PCR probes (87.7%) identified subsets of clones found by overgo hybridization. This supports the *in silico *association data. The BAC data from 55 out of 81 (68%) PCR-markers successfully anchored BACs to genetic positions in contrast to the unsuccessful anchoring of all 81 markers based on overgo data alone.

## Discussion

We describe an approach combining a BAC pooling strategy with PCR-based primer screening that can aid in physical map construction and provide anchor points to the genetic map. This methodology allows identification of overlapping BAC clones while simultaneously establishing links between the BAC contigs and the genetic map. The advantages of screening BAC pool DNAs with PCR-based primers include a low rate of false positives, low cost, and increased throughput compared to conventional hybridization techniques. Although the use of this BAC pooling strategy for integrated map construction was successfully demonstrated by Klein et al[12] in sorghum, our study is the first report to show that BAC pooling can be used for an organism with a larger genome and highly repetitive DNA. In addition, the number of clones per pool reported here is higher than the pools used in other organisms: 1,920 clones/pool (one-eleventh genome equivalent) in human [18] and 1,024 or 768 clones/pool (one-fifth or one-seventh genome equivalent) in sorghum [14]. The six-dimensional pools also represent an alternative to the use of radiation hybrids for physical mapping. Radiation hybrids have been constructed in maize however their utility for fine scale mapping is limited because of low resolution and marker retention frequencies [18,19]; problems which are not present in the BAC pools described here.

The average number of BAC per marker was lower than expected, 4.68 vs. 6.0. This could be explained by; 1) lack of product amplification in one or more dimensions of the 6 pools (deconvolution requires amplification in all six dimensions), 2) failure to identify all clones of highly represented sequences due to occlusion, 3) no representation of the region the marker was designed from in the *Hin*dIII BAC library, 4) absence of the locus within the subset of the BAC library used to construct the BAC pools, or 5) over estimation of representation in the BAC library.

One hundred forty-nine of the 237 (62.9%) markers with genetic map positions identified BACs assembled into single contigs providing unambiguous anchoring points. Forty-two of the 55 markers that identified BACs assembled in more than one contig also anchor contigs to the genetic map. In total, 191 markers anchored physical contigs to their genetic positions demonstrating the utility of the BAC pools as a powerful method to integrate the physical and genetic maps and to resolve conflicts caused by other anchoring methods. The results are displayed on iMap [1] and WebFPC [2] as a public resource.

The BAC pools were also used to aid in refining marker order for closely linked genes on the genetic map, rapid mapping of gene families or duplicated genomic regions, and inferring positions of monomorphic markers by assigning to anchored contigs. Furthermore, physical mapping of monomorphic markers allow us to infer the genetic map locations for these markers based on the genetic location of the linked markers from each BAC contig. Our preliminary results using markers with small genetic distances between them suggest that the relationships between genetic distance in centiMorgans and physical distance in base pairs are not always parallel due to the difference in frequency of recombination along the chromosome. Arabidopsis and rice studies reported similar observations [25,26]. The relationship between physical distance and genetic recombination can vary widely due to difference in recombination rates along the chromosome. Minor genotyping errors can also cause incorrect marker order in small segments of the genetic map due to lack of informative recombination events. Positional cloning studies rely heavily on closely linked markers that have unambiguous map orders. Our results demonstrate that the maize BAC pools offer a powerful tool for resolving gene in low recombination regions. In addition, although the local orders of markers are slightly different in the physical map compared to the genetic map, overall our results confirm the robustness and high-resolution of the IBM genetic map.

The data also demonstrates that the pools can be used for locus-specific contig assembly of duplicated genes, which is difficult using hybridization-based method. This information provides a significant aid to positional cloning of duplicated loci, to understanding biochemical networking and/or regulation between pathways, and for functional study of these transcription factors.

The data from 81 PCR-based markers associated with overgo probes by *in silico *methods allowed resolution of prior conflicts for 88% of the loci. However, the discrepancies caused by the rest of the data (12%) suggest that, if ties between the physical and genetic maps are solely established by *in silico *connections and are contradictory with the pool data, then data produced from the pools should be given preference in anchoring contigs due to the low rate of false positives compared to hybridization-based approaches.

The pools provide a fast, efficient, cost-effective way to anchor contigs. The entire process from PCR to deconvolution of data can be completed in a day. Only 288 PCR reactions per primer are needed to screen 110,592 BAC clones simultaneously at a cost of about $100 per locus including labor. PCR-based screening of the DNA pools provides anchoring points more quickly and more unambiguously than with traditional hybridization methods. Approximately 2500 additional genetic markers (the number of genetic markers currently mapping directly on the IBM genetic mapping population) will be needed to generate a completely integrated map for the maize genome. The BAC pools will be screened with the rest of the publicly available mapped SSR markers and with single-copy RFLP clones to achieve this goal. Once a comprehensive anchored physical map is available the BAC pools represent a robust, economical method to map genes or new sequences to the integrated map.

## Conclusion

The six-dimensional maize BAC pools were successful in assigning markers to BAC despite the large genome size and amount of repetitive DNA present. This suggests that a similar strategy can be employed in other organisms with similar genome structure. Our data indicate that in addition to anchoring the genetic and physical maps, the pools can be used to define local marker order, resolve conflicting hybridization-based marker: BAC assignments, and rapidly map duplicate factors or members of a gene family. Use of the pools to obtain additional marker: BAC associations in maize could speed assembly and validation of the fingerprint-based physical map.

## Methods

### BAC library

A maize *Hin*dIII BAC library was constructed using inbred B73 at the Clemson University Genomic Institute (CUGI) [27]. This library has an average insert size of 136 Kb and a genome coverage of 13.5×. A previous study demonstrated that the maize *Hin*dIII BAC library provides adequate coverage for maize genome research [11]. A subset of the first 110,592 clones was used for the pooling, providing coverage of 6× genome equivalents.

### BAC pooling strategy

#### Cube Design

Two hundred eighty-eight 384-well microtiter plates were arranged into a cube containing 48 layers (or plates) with six plates per layer for the BAC pooling strategy. The six plates in a layer were arranged in a 3 × 2 pattern. Because each 384-well plate is an array of 16 rows and 24 columns of wells, this 3 × 2 pattern resulted in each layer of the cube containing wells in a 48 row × 48 column array (2,304 clones/layer).

Every well in the cube has a unique address defined by its *x*, *y*, and *z *coordinates relative to the axes of the cube (Fig. 1). Although the assignment of *x*, *y*, and *z *to a particular axis is arbitrary, for our purposes, the *x*-axis extends left to right, as seen by an observer examining one side of the stack. A rank of wells parallel to the *x*-axis is a row. The *y*-axis extends away from the observer towards the horizon. A rank of wells parallel to the *y*-axis is a column. Finally, the *z*-axis is mutually perpendicular to *x*- and *y*-axes and defines vertical position (plate or layer number) within the cube. The origin of the cube is defined as the far-left corner of the topmost layer with its coordinates being 1,1,1.

#### Pooling Strategy

BAC pools were produced by sampling the cube of stacked microtiter plates in six different matrices (Fig. 1). Each pool represents the intersection of a plane with the cube. Plate pools (PP) were prepared from each layer or plate of the cube. All BACs in a plate pool share the same layer (*z*) coordinate. The front surface of the stack facing an observer was termed the *face*. Planes parallel to this surface defined face pools (FP). Each face pool consists of BACs that shares the same *y*-coordinate. The surfaces left and right of the stack were termed *sides*. Planes parallel to these surfaces defined side pools (SP). Each side pool consists of BACs that share the same *x*-coordinate. The three remaining pool types were taken at a diagonal angle through the stack. Row pools (RP) were established by row R (*y*) plus plate P (*z*). Column pools (CP) were established in the same manner as row pools by column C (*x*) added to plate P (*z*). All wells from row R (*y*) and column C (*x*) consisted of diagonal pools (DP). To keep the number of wells in each row, column, and diagonal pool constant (2,304 clones per pool), wrapping occurred. That is, when the added value from RP, CP, and DP was greater than 48 (the longest of the 3 dimensions, *x*, *y*, and *z*, used in the stack), then 48 was subtracted from the added value to give the correct pool number and equal number of clones per pool (Fig. 2C).

### BAC pool DNA isolation

Each time one of the six pool types was prepared, the 288 384-well microtiter plates comprising the pooling stack were inoculated with BAC stocks. BACs were inoculated from a frozen stock plate using a 384-well pin tool into microtiter plates containing 70 μl LB media plus 12.5 μg/ml chloramphenicol per well and the plates were incubated overnight at 37 °C. The next day, another set of 288 384-well microtiter plates with TB media plus 12.5 μg/ml chloramphenicol were inoculated with the BACs from the prior night's LB plates and grown overnight using the same procedure as the day before. The third day the plates were arranged into the cube and sampled in one of the six matrices to generate a particular pool type. For BAC pooling, 45 μl of culture was removed from each well using 16- or 12-channel multi-pipette for five (plate, face, side, row, and column) matrices and a single channel pipette for the diagonal matrix. The BAC cultures were placed in sterile containers with each container defining a given pool. BAC DNA isolation was performed using the procedure described by Klein et al. [14] with slight modifications [see additional file 1].

### PCR-based screening of BAC pools

Gene-specific primers for transcription factors and Unigene clusters were designed using the Primer3 application from the Whitehead Institute (Cambridge, MA). The primer design conditions were *T*m 60–65 °C, target 63 °C with the difference in the *T*m of the two primers less than 1 °C, minimum primer length 18 nt, maximum primer length 24 nt, and target of 22 nt. Primers were checked for dimerization and hairpin loop formation using IDT's oligo analyzer 2.5 [28]. Primer sequences that passed selection criteria were analyzed by BLAST analysis against the NCBI non-redundant database for homology to other sequences. Primers were synthesized by Sigma-Genosys (The Woodlands, Texas).

Several groups of PCR primers were used in this study, including previously developed SSRs [29] and newly designed gene-specific primers (see Additional File 2). Monomorphic SSR markers were provided by Dr. Michael McMullen at the University of Missouri (Columbia, MO). These SSRs were monomorphic based on screening against 11 U.S. maize inbreds, five maize landraces, and six teosintes, and, thus are considered genetically unmappable.

All PCR reactions were performed using a Mastercycler (Eppendorf Scientific Inc., Westbury, NY) or a Tetrad thermal cycler (MJ Research Inc., Waltham, MA). PCR reactions were performed with a 'touchdown' profile with slightly modified cycle number, between 20 and 30, from the standard SSR PCR protocol and reduced amount of template DNA (5 ng) and primers (10 ng each forward and reverse primer) per reaction [30]. The PCR products were resolved on 4.5% Super Fine Resolution (SFR) agarose (Amresco, Solon, OH), 1 × TBE gels containing ~ 2 μg/ml ethidium bromide. SSR gel images were digitized using an AlphaImager 2200 (Alpha Innotech Corp., San Leandro, CA) and scored three times by independent readers.

### Data deconvolution

The Resolve Script software developed to deconvolute the sorghum pool data [31] was modified to accommodate our pool dimensions. The program is written in Perl. Resolve script provides a list of candidate BAC clones from the positive pool data obtained with primer pairs. LabConvert, a visual basic application [32], further analyzed this output. LabConvert identifies the BAC addresses in the particular plate location and provides the necessary input file format for the FPC assembly software. Contig assignments were made based on the WebFPC V7.2 assembly from July 19, 2005 [33].

### Error reduction

The pooled BAC libraries were deliberately over-sampled (6× genome equivalents) to ensure a high probability of retrieving every maize DNA sequence. A conventional pooling strategy using only three dimensions was considered inadequate to unambiguously identify an individual BAC responsible for a PCR signal because of the possibility of data occlusion. Although there are six pool types, there are only three degrees of freedom (*x*, *y*, *z*) in the pooling system. The redundancy makes it possible to use the presence of a signal (i.e., a PCR amplification product) in two pool types to predict the presence of the same signal in a third pool type. Specifically, (1) plate pools and face pools predict row pools, (2) plate pools and side pools predict column pools, and (3) face pools and side pools predict diagonal pools. The process eliminates many alternative addresses and requires that a signal must be detected multiple times in the appropriate pools before it is considered meaningful. This feature greatly reduces the frequency of false-positives but increases the rate of false-negatives. To minimize the rate of false-negatives, the Resolve script output was thoroughly examined for markers where the number of resolved positives was lower than half the expected number of minimum positives. Gel images of these markers were crosschecked to verify that all six dimensions amplified a similar number of PCR products. Dimensions with an exceptionally low number of products were amplified again.

## Authors' contributions

YSY participated in the design of the experiment, carried out the pool construction, data deconvolution, and drafted the manuscript. PM carried out the pool construction and PCR assays. HSV carried out bioinformatic analysis. TAM participated in pool construction. PC participated in pool construction. PEK participated in design of the experiment. JEM participated in design of the experiment. MDM participated in design of the monomorphic marker assays. ZF carried out bioinformatics analysis. MLS carried out bioinformatics related to map integration. JMG participated in pool construction. EHC participated in map integration. GLD participated in design of the experiment, carried out pool construction, contributed to data interpretation and revision of the manuscript.

## Supplementary Material

Additional File 2 Supplementary Table 1.  Marker to BAC and BAC to contig associations made using 288 six-dimensional  BAC pools.

Additional File 1 BAC pool DNA isolation

## Figures and Tables

**Table 1:** SSR markers with a low recombination rate on the IBM genetic map, using the physical map to redefine their order

Bin	Map coordinate	SSR markers	GenBank Accession #	Contigs hit (#of BACs hit within contig)
1.05	467.0*	p-umc1906	B68068	**33**(3)
	467.9	p-umc1903	G68065	**33 **(6)
3.04	190.6	p-bnlg1019	-	*116*(5)
	190.8	p-umc1717	AW573521	**117 **(1)
	190.8*	p-bnlg1452	-	*116 *(2)
	190.8*	p-bnlg1113	-	**117 **(4)
	191.1	p-umc1655	AZ916071	*116 *(5)
5.06	492.6	p-umc1941	-	**250**(4)
	493.5*	p-umc1680	AW261378	**250 **(1)
	493.5*	p-umc1524	AQ844589	249 (2)
	493.7	p-phi087	-	**250 **(4)
	500.1*	p-umc2306	BI431083	**250 **(4)
	500.7*	p-bnlg609	-	**250 **(7)
6.04	181.9*	p-umc65	G10860	**276 **(2)
	189.5*	p-umc1796	AW562820	b0218P06^a^
	189.9	p-umc1918	G68080	**276 **(5)
7.03	405.5*	p-umc1301	AI947719	**322 **(7)
	405.5*	p-umc1936	G68098	**322 **(2)

^a^p-umc1796 identified a single BAC clone, b0218P06. This clone was not fingerprinted and thus not displayed in WebFPC data. * on-frame position on the map.

**Table 2:** Transcription factors used for PCR primer development and BAC pools screening

Transcription Factor	Map bin location	Functions	Myb or Myc	PCR marker	Copy #	Contig # (# of BACs hit)	GenBank Accession
*C1*	9.01	Anthocyanin regulation; Transcription factor	Myb	p-C1	2	370 (2)	M37153
*PL1*	6.04*	Anthocyanin regulation; Transcription factor	Myb	p-phi031, p-umc1014	2	**280 **(3), 386 (1) **280 **(3)	L19495
*P1*	1.03*	Anthocyanin regulation; Transcription factor	Myb	p-phi095	-	11 (6), 210 (1)	Z11879
*MRP1 (= ZmCMT) Myb related protein*	8.05	*MRP1 *gene; one repeat myb transcriptional factor; first transfer cell specific TF	Myb	p-MRP1	1	32 (4), 354 (1)	AJ318518
*R2R3*	-*	Regulation of secondary metabolism, control of cellular morphogenesis and regulation of meristem formation	Myb	p-R2R3	High	265 (2)	AF474115
*O2 (opaque 2)*	7.01*	DNA-binding protein; leucine zipper; *opaque 2 *gene; transcriptional activator. Regulates zein deposition in maize endosperm	Myb	p-Phi112, p-umc1066	-	**296 **(3) **296 **(2)	X16618
*Myb2*	3.05	Similar to Arabidopsis *cdc5 *myb protein	Myb	p-Myb2	2	131 (5)	T18844
*R1*	10.06	Anthocyanin regulation; Transcription factor	Myc	p-R1	-	415 (7)	U93178
*SN1-bol3*	10.06	Anthocyanin regulation; SN gene; Transcription factor	Myc	p-Sn1	-	415 (6), 421 (1)	X60706
*B*	2.03*	Anthocyanin regulation; B transcriptional activator; Transcriptional activator	Myc	p-umc1776	-	74 (6)	X70790
*Myc7E*	-	Regulate iron metabolism in both low- and high-affinity iron transport; Transcription factor	Myc	p-Myc7	-	26 (3)	AF061107
*ZmHOX1 (Homeobox1)*	8.05	Protein product binds to feedback control element of *sh1 *promoter	N.A.^a^	p-umc1309, p-umc1343	1	325 (1), **353 **(3) 96 (1), 110 (1), 145 (1), **353 **(5)	-
*MBF1*	4.05, 9.03	Transcriptional factor	N.A.^a^	p-MBF1	2	162 (1)	T14752
*goliath1*	4.08	Transcription factor, similar to Drosophila Goliath protein	N.A.^a^	p-gol1	1	184 (1)	T18797
*Golden plant2 (g2)*	3.00*	putative transcription factor *golden2 *gene	N.A.^a^	p-umc2109	-	111 (7)	-

^a^N.A.: not available. *Anchor marker.
