# Supplementary material for: A BAC pooling strategy combined with PCR-based screenings in a large, highly repetitive genome enables integration of the maize genetic and physical maps
Source: BMC Genomics. 2007 Feb 9;8:47. doi: 10.1186/1471-2164-8-47 (PMC1821331; doi:10.1186/1471-2164-8-47)
Supplement: Additional File 2 — Supplementary Table 1. Marker to BAC and BAC to contig associations made using 288 six-dimensional BAC pools. [file 1471-2164-8-47-S2.doc]

Supplementary Table 1. Marker to BAC and BAC to contig associations made using 288 six-dimensional BAC pools.

| **Marker** | **BAC clone** | **Contig** |
| --- | --- | --- |
| bnlg1014 | b0036L14 | ctg362 |
| bnlg1014 | b0138L14 | ctg004 |
| bnlg1014 | b0074A07 | ctg004 |
| bnlg1014 | b0182A10 |  |
| bnlg1014 | b0092D02 | ctg004 |
| bnlg1014 | b0008N23 |  |
| bnlg1014 | b0284B14 | ctg004 |
| bnlg1018 | b0044L19 | ctg078 |
| bnlg1018 | b0174G05 | ctg062 |
| bnlg1018 | b0172L05 | ctg078 |
| bnlg1018 | b0167I02 | ctg078 |
| bnlg1018 | b0232J05 |  |
| bnlg1018 | b0228F06 | ctg078 |
| bnlg1025 | b0051J03 |  |
| bnlg1025 | b0061L10 | ctg046 |
| bnlg1025 | b0009H09 | ctg046 |
| bnlg1031 | b0074E04 | ctg363 |
| bnlg1031 | b0019M16 | ctg363 |
| bnlg1031 | b0056P06 |  |
| bnlg1031 | b0089B11 | ctg363 |
| bnlg1031 | b0101O02 |  |
| bnlg1035 | b0017J11 | ctg125 |
| bnlg1035 | b0246C17 |  |
| bnlg1035 | b0261C04 | ctg125 |
| bnlg1035 | b0246C16 |  |
| bnlg1035 | b0241O15 |  |
| bnlg1036 | b0030N24 | ctg091 |
| bnlg1036 | b0032N18 | ctg091 |
| bnlg1036 | b0192C06 | ctg091 |
| bnlg1046 | b0143N19 | ctg211 |
| bnlg1046 | b0080O16 | ctg211 |
| bnlg1046 | b0003N12 | ctg211 |
| bnlg1046 | b0173C09 | ctg211 |
| bnlg1046 | b0098D13 | ctg211 |
| bnlg1063 | b0063H14 |  |
| bnlg1063 | b0069D15 |  |
| bnlg1063 | b0085E07 | ctg134 |
| bnlg1063 | b0198O13 |  |
| bnlg1063 | b0082H24 | ctg134 |
| bnlg1063 | b0158C05 | ctg134 |
| bnlg1063 | b0244E05 | ctg134 |
| bnlg1064 | b0169E13 | ctg074 |
| bnlg1064 | b0056N13 |  |
| bnlg1064 | b0244A24 | ctg074 |
| bnlg1079 | b0014A02 |  |
| bnlg1079 | b0164G04 | ctg262 |
| bnlg1079 | b0075G04 | ctg057 |
| bnlg1079 | b0053A03 |  |
| bnlg1079 | b0052K02 | ctg106 |
| bnlg1079 | b0047A09 |  |
| bnlg1079 | b0122A15 | ctg096 |
| bnlg1079 | b0045K06 | ctg402 |
| bnlg1079 | b0138L15 | ctg402 |
| bnlg1079 | b0251F23 | ctg402 |
| bnlg1079 | b0265D16 | ctg402 |
| bnlg1079 | b0247L23 | ctg220 |
| bnlg1094 | b0093B22 | ctg302 |
| bnlg1094 | b0022D21 |  |
| bnlg1094 | b0209D10 |  |
| bnlg1113 | b0127L11 |  |
| bnlg1113 | b0165E09 | ctg117 |
| bnlg1113 | b0131L04 | ctg117 |
| bnlg1113 | b0170B01 | ctg117 |
| bnlg1113 | b0053A24 |  |
| bnlg1113 | b0157L11 | ctg117 |
| bnlg1113 | b0055I11 |  |
| bnlg1113 | b0090C09 |  |
| bnlg1118 | b0127H05 |  |
| bnlg1118 | b0046A20 | ctg253 |
| bnlg1118 | b0129C05 | ctg253 |
| bnlg1118 | b0002L01 |  |
| bnlg1118 | b0098M09 | ctg253 |
| bnlg1129 | b0146F20 |  |
| bnlg1129 | b0033E24 |  |
| bnlg1129 | b0019F05 | ctg391 |
| bnlg1129 | b0036A24 |  |
| bnlg1129 | b0074K14 | ctg391 |
| bnlg1129 | b0074K20 | ctg036 |
| bnlg1129 | b0093L23 |  |
| bnlg1129 | b0232D19 | ctg391 |
| bnlg1137 | b0114J24 |  |
| bnlg1137 | b0181P18 |  |
| bnlg1137 | b0176A02 | ctg182 |
| bnlg1137 | b0077P06 |  |
| bnlg1144 | b0069B06 | ctg111 |
| bnlg1144 | b0199B10 | ctg111 |
| bnlg1144 | b0139N10 | ctg111 |
| bnlg1144 | b0241H12 |  |
| bnlg1152 | b0076I04 | ctg325 |
| bnlg1152 | b0186E01 | ctg388 |
| bnlg1152 | b0004I24 | ctg361 |
| bnlg1152 | b0258K04 | ctg361 |
| bnlg1152 | b0232J03 | ctg361 |
| bnlg1152 | b0243J15 |  |
| bnlg1152 | b0236D09 |  |
| bnlg1154 | b0103P16 | ctg281 |
| bnlg1154 | b0065D09 |  |
| bnlg1154 | b0059D14 | ctg032 |
| bnlg1154 | b0171A14 | ctg281 |
| bnlg1154 | b0191D15 | ctg281 |
| bnlg1154 | b0182M08 | ctg281 |
| bnlg1154 | b0056F20 |  |
| bnlg1154 | b0247I01 | ctg281 |
| bnlg1159 | b0038D17 |  |
| bnlg1159 | b0071F19 |  |
| bnlg1159 | b0136K05 | ctg382 |
| bnlg1159 | b0071F19 |  |
| bnlg1159 | b0175N22 |  |
| bnlg1159 | b0055B17 | ctg382 |
| bnlg1159 | b0055B17 | ctg382 |
| bnlg1159 | b0038D17 |  |
| bnlg1159 | b0175N22 |  |
| bnlg1159 | b0130E09 | ctg382 |
| bnlg1159 | b0136K05 | ctg382 |
| bnlg1159 | b0051D05 |  |
| bnlg1159 | b0070E08 |  |
| bnlg1159 | b0070E08 |  |
| bnlg1159 | b0130E09 | ctg382 |
| bnlg1159 | b0051D05 |  |
| bnlg1159 | b0246M05 |  |
| bnlg1159 | b0265L21 |  |
| bnlg1159 | b0265L21 |  |
| bnlg1159 | b0246M05 |  |
| bnlg1160 | b0139P24 | ctg138 |
| bnlg1160 | b0078G21 |  |
| bnlg1175 | b0122F02 | ctg078 |
| bnlg1175 | b0198H20 | ctg078 |
| bnlg1175 | b0021P23 |  |
| bnlg1175 | b0162O16 | ctg078 |
| bnlg1175 | b0183O19 |  |
| bnlg1175 | b0247M02 | ctg078 |
| bnlg1175 | b0258P01 |  |
| bnlg1175 | b0252G11 | ctg078 |
| bnlg1176 | b0116J04 | ctg049 |
| bnlg1176 | b0048C14 | ctg004 |
| bnlg1176 | b0108N14 | ctg023 |
| bnlg1176 | b0145E20 | ctg117 |
| bnlg1176 | b0007C02 |  |
| bnlg1176 | b0103I16 |  |
| bnlg1176 | b0217M12 | ctg123 |
| bnlg1176 | b0221I12 | ctg231 |
| bnlg1176 | b0270A10 |  |
| bnlg1176 | b0222C23 | ctg090 |
| bnlg1176 | b0267B04 | ctg028 |
| bnlg1176 | b0269N02 | ctg172 |
| bnlg1176 | b0257N02 |  |
| bnlg1185 | b0063F05 | ctg419 |
| bnlg1185 | b0166J07 | ctg419 |
| bnlg1185 | b0111G21 | ctg419 |
| bnlg1185 | b0065M18 | ctg419 |
| bnlg1185 | b0251M24 |  |
| bnlg1189 | b0135B11 | ctg182 |
| bnlg1189 | b0160H21 | ctg182 |
| bnlg1189 | b0255B01 | ctg182 |
| bnlg1189 | b0210I02 |  |
| bnlg1203 | b0138M08 | ctg011 |
| bnlg1203 | b0120P12 | ctg011 |
| bnlg1203 | b0040J09 | ctg011 |
| bnlg1203 | b0146E08 | ctg011 |
| bnlg1203 | b0077I23 |  |
| bnlg1203 | b0288H18 | ctg011 |
| bnlg1208 | b0093P24 |  |
| bnlg1208 | b0019K08 |  |
| bnlg1208 | b0227B18 |  |
| bnlg1247 | b0195B08 | ctg008 |
| bnlg1247 | b0159J08 |  |
| bnlg1247 | b0064G19 |  |
| bnlg1247 | b0027G05 |  |
| bnlg1247 | b0046H14 | ctg304 |
| bnlg1247 | b0115P04 |  |
| bnlg1265 | b0086B10 |  |
| bnlg1265 | b0229L04 |  |
| bnlg1265 | b0259I07 | ctg164 |
| bnlg1267 | b0140B19 | ctg103 |
| bnlg1267 | b0073L07 | ctg103 |
| bnlg1267 | b0128C04 | ctg103 |
| bnlg1267 | b0147L14 | ctg103 |
| bnlg1267 | b0074L04 |  |
| bnlg1267 | b0278E11 | ctg103 |
| bnlg1267 | b0249G23 | ctg103 |
| bnlg1297 | b0241A13 |  |
| bnlg1297 | b0228K16 |  |
| bnlg1297 | b0224D16 |  |
| bnlg1297 | b0261E22 |  |
| bnlg1316 | b0020F21 | ctg104 |
| bnlg1316 | b0089D21 | ctg104 |
| bnlg1316 | b0191N11 | ctg104 |
| bnlg1316 | b0010H15 | ctg104 |
| bnlg1316 | b0285G02 |  |
| bnlg1327 | b0193N21 |  |
| bnlg1327 | b0118D04 | ctg071 |
| bnlg1327 | b0075I23 | ctg071 |
| bnlg1327 | b0154O12 | ctg071 |
| bnlg1329 | b0189G02 | ctg098 |
| bnlg1331 | b0152H15 | ctg054 |
| bnlg1331 | b0013P13 |  |
| bnlg1331 | b0103C13 | ctg054 |
| bnlg1331 | b0001O19 | ctg054 |
| bnlg1331 | b0013O13 | ctg054 |
| bnlg1331 | b0037F11 |  |
| bnlg1331 | b0231C24 | ctg054 |
| bnlg1360 | b0222H17 |  |
| bnlg1367 | b0165K20 | ctg293 |
| bnlg1367 | b0174D16 | ctg293 |
| bnlg1367 | b0024J16 | ctg293 |
| bnlg1367 | b0273P09 |  |
| bnlg1367 | b0275M07 | ctg293 |
| bnlg1370 | b0112H21 |  |
| bnlg1370 | b0191F12 | ctg155 |
| bnlg1370 | b0196B07 | ctg155 |
| bnlg1370 | b0234L01 |  |
| bnlg1371 | b0003O03 |  |
| bnlg1371 | b0163E19 | ctg262 |
| bnlg1371 | b0146O03 |  |
| bnlg1371 | b0069C19 | ctg262 |
| bnlg1371 | b0206J03 |  |
| bnlg1371 | b0259A09 | ctg262 |
| bnlg1371 | b0229C15 | ctg262 |
| bnlg1371 | b0222E14 |  |
| bnlg1371 | b0265J17 | ctg331 |
| bnlg1371 | b0217H09 | ctg262 |
| bnlg1380 | b0126P10 | ctg304 |
| bnlg1380 | b0092B11 | ctg304 |
| bnlg1380 | b0095P22 |  |
| bnlg1380 | b0133C17 |  |
| bnlg1380 | b0142O24 | ctg304 |
| bnlg1380 | b0223G22 | ctg304 |
| bnlg1401 | b0174E11 | ctg373 |
| bnlg1401 | b0049N01 | ctg373 |
| bnlg1429 | b0066O14 |  |
| bnlg1429 | b0067B16 |  |
| bnlg1429 | b0055O14 | ctg007 |
| bnlg1434 | b0145G14 | ctg154 |
| bnlg1434 | b0019K03 | ctg154 |
| bnlg1434 | b0136F15 | ctg154 |
| bnlg1434 | b0256B19 |  |
| bnlg1434 | b0206O11 | ctg154 |
| bnlg1434 | b0283E21 | ctg154 |
| bnlg1434 | b0224G02 | ctg154 |
| bnlg1450 | b0131F11 | ctg419 |
| bnlg1450 | b0168C07 |  |
| bnlg1450 | b0025B11 | ctg419 |
| bnlg1450 | b0251O07 | ctg419 |
| bnlg1452 | b0133D15 | ctg116 |
| bnlg1452 | b0169A15 | ctg233 |
| bnlg1452 | b0241B09 | ctg116 |
| bnlg1452 | b0268H23 |  |
| bnlg1484 | b0197I19 | ctg011 |
| bnlg1484 | b0015H07 | ctg011 |
| bnlg1484 | b0179C12 | ctg011 |
| bnlg1496 | b0126G20 | ctg150 |
| bnlg1496 | b0181C14 |  |
| bnlg1496 | b0002F06 | ctg150 |
| bnlg1496 | b0286F16 | ctg150 |
| bnlg1496 | b0209E15 | ctg150 |
| bnlg1496 | b0273A22 | ctg150 |
| bnlg1496 | b0241H03 | ctg150 |
| bnlg1496 | b0219E20 | ctg150 |
| bnlg1556 | b0143P15 | ctg044 |
| bnlg1556 | b0123I07 |  |
| bnlg1556 | b0281A21 | ctg044 |
| bnlg1598 | b0086P02 |  |
| bnlg1598 | b0106H22 | ctg270 |
| bnlg1598 | b0064D23 | ctg038 |
| bnlg1598 | b0146P24 |  |
| bnlg1598 | b0104J22 |  |
| bnlg1598 | b0112H16 | ctg038 |
| bnlg1598 | b0006H02 | ctg038 |
| bnlg1598 | b0112G20 | ctg038 |
| bnlg1598 | b0210A10 |  |
| bnlg1598 | b0277P24 |  |
| bnlg1599 | b0011H10 |  |
| bnlg1599 | b0112K20 | ctg354 |
| bnlg1599 | b0139I20 | ctg354 |
| bnlg1599 | b0051C21 | ctg354 |
| bnlg1601 | b0091N14 | ctg124 |
| bnlg1601 | b0182D24 |  |
| bnlg1601 | b0098D04 |  |
| bnlg1601 | b0045A14 | ctg124 |
| bnlg1601 | b0100J13 | ctg124 |
| bnlg1601 | b0175P06 | ctg124 |
| bnlg1601 | b0192P21 |  |
| bnlg1601 | b0201J09 |  |
| bnlg1605 | b0128M10 |  |
| bnlg1605 | b0174L18 |  |
| bnlg1605 | b0190J20 | ctg141 |
| bnlg1605 | b0265A06 | ctg141 |
| bnlg1605 | b0263K02 | ctg141 |
| bnlg1641 | b0154D23 |  |
| bnlg1641 | b0179D15 | ctg266 |
| bnlg1641 | b0108J04 | ctg266 |
| bnlg1641 | b0179D15 | ctg266 |
| bnlg1641 | b0073O21 | ctg266 |
| bnlg1641 | b0154D23 |  |
| bnlg1641 | b0108J04 | ctg266 |
| bnlg1641 | b0073O21 | ctg266 |
| bnlg1641 | b0233A06 |  |
| bnlg1641 | b0233A06 |  |
| bnlg1647 | b0136E02 | ctg112 |
| bnlg1647 | b0043A21 | ctg112 |
| bnlg1647 | b0198O07 |  |
| bnlg1647 | b0031F18 |  |
| bnlg1647 | b0129L01 | ctg112 |
| bnlg1647 | b0265O23 |  |
| bnlg1647 | b0207D03 | ctg112 |
| bnlg1647 | b0283J11 | ctg112 |
| bnlg1651 | b0031J23 | ctg354 |
| bnlg1651 | b0088P23 | ctg354 |
| bnlg1651 | b0123P08 |  |
| bnlg1651 | b0016L12 | ctg354 |
| bnlg1651 | b0029E11 | ctg354 |
| bnlg1651 | b0017N22 | ctg354 |
| bnlg1651 | b0067B22 |  |
| bnlg1651 | b0286E06 | ctg354 |
| bnlg1655 | b0156D17 | ctg405 |
| bnlg1655 | b0168I24 | ctg408 |
| bnlg1655 | b0097M13 |  |
| bnlg1655 | b0177O23 |  |
| bnlg1655 | b0158N20 | ctg405 |
| bnlg1655 | b0051K24 | ctg405 |
| bnlg1655 | b0234N11 |  |
| bnlg1671 | b0178F22 | ctg235 |
| bnlg1671 | b0145P18 | ctg299 |
| bnlg1671 | b0178F06 | ctg057 |
| bnlg1671 | b0147A03 | ctg057 |
| bnlg1671 | b0217P18 | ctg057 |
| bnlg1671 | b0284G19 | ctg057 |
| bnlg1677 | b0085K02 |  |
| bnlg1677 | b0123G23 | ctg417 |
| bnlg1677 | b0015O14 |  |
| bnlg1677 | b0181B11 | ctg417 |
| bnlg1677 | b0134N06 | ctg417 |
| bnlg1677 | b0127K05 | ctg417 |
| bnlg1677 | b0243G15 |  |
| bnlg1677 | b0211C20 | ctg417 |
| bnlg1677 | b0259N23 | ctg417 |
| bnlg1702 | b0251O18 |  |
| bnlg1712 | b0040A13 | ctg404 |
| bnlg1712 | b0184F13 | ctg404 |
| bnlg1712 | b0017G13 | ctg404 |
| bnlg1712 | b0080G10 | ctg404 |
| bnlg1712 | b0083P13 | ctg404 |
| bnlg1712 | b0002G14 | ctg404 |
| bnlg1712 | b0040F13 | ctg108 |
| bnlg1712 | b0038G10 | ctg336 |
| bnlg1712 | b0065G20 |  |
| bnlg1712 | b0101A11 | ctg404 |
| bnlg1712 | b0271C24 | ctg404 |
| bnlg1732 | b0169B04 | ctg255 |
| bnlg1732 | b0110A01 | ctg287 |
| bnlg1732 | b0063F01 | ctg287 |
| bnlg1732 | b0227P03 |  |
| bnlg1732 | b0258K22 | ctg173 |
| bnlg1732 | b0268M06 | ctg287 |
| bnlg1732 | b0255L14 | ctg287 |
| bnlg1740 | b0110D14 | ctg289 |
| bnlg1740 | b0098F16 |  |
| bnlg1740 | b0096F15 | ctg289 |
| bnlg1740 | b0107L11 | ctg289 |
| bnlg1740 | b0177E11 |  |
| bnlg1740 | b0220K10 | ctg289 |
| bnlg1740 | b0230G09 | ctg235 |
| bnlg1746 | b0126M02 | ctg105 |
| bnlg1746 | b0126M02 | ctg105 |
| bnlg1746 | b0260J16 | ctg105 |
| bnlg1746 | b0260J16 | ctg105 |
| bnlg1754 | b0188A20 |  |
| bnlg1759 | b0133C10 | ctg117 |
| bnlg1759 | b0075E05 |  |
| bnlg1759 | b0093P15 |  |
| bnlg1759 | b0133B08 | ctg289 |
| bnlg1759 | b0184B16 | ctg289 |
| bnlg1759 | b0133B10 | ctg235 |
| bnlg1805 | b0089J07 | ctg322 |
| bnlg1805 | b0029J21 | ctg322 |
| bnlg1805 | b0052H14 | ctg322 |
| bnlg1805 | b0138K23 | ctg322 |
| bnlg1805 | b0086K15 | ctg322 |
| bnlg1805 | b0027E21 |  |
| bnlg1805 | b0250H14 | ctg066 |
| bnlg1805 | b0251H12 | ctg322 |
| bnlg1808 | b0014M11 |  |
| bnlg1808 | b0088M03 |  |
| bnlg1810 | b0124H18 | ctg369 |
| bnlg1810 | b0171F11 |  |
| bnlg1810 | b0138K18 | ctg369 |
| bnlg1810 | b0092K09 | ctg369 |
| bnlg1810 | b0237N02 | ctg369 |
| bnlg1810 | b0246K18 |  |
| bnlg1810 | b0231D23 | ctg369 |
| bnlg1810 | b0246K03 |  |
| bnlg1810 | b0235K23 | ctg118 |
| bnlg1811 | b0055M03 |  |
| bnlg1811 | b0119F16 | ctg017 |
| bnlg1811 | b0170B20 |  |
| bnlg1811 | b0087O10 |  |
| bnlg1811 | b0207D13 | ctg017 |
| bnlg1823 | b0148P23 |  |
| bnlg1823 | b0157G21 | ctg363 |
| bnlg1823 | b0144E11 | ctg363 |
| bnlg1823 | b0095F11 | ctg363 |
| bnlg1823 | b0030D12 | ctg363 |
| bnlg1823 | b0219I06 | ctg363 |
| bnlg1823 | b0221K06 | ctg241 |
| bnlg1831 | b0074L09 | ctg091 |
| bnlg1831 | b0002B24 | ctg091 |
| bnlg1831 | b0046I15 |  |
| bnlg1831 | b0167E22 | ctg091 |
| bnlg1831 | b0204A15 | ctg091 |
| bnlg1831 | b0257J04 | ctg091 |
| bnlg1831 | b0205P03 | ctg091 |
| bnlg1834 | b0109I03 | ctg335 |
| bnlg1834 | b0198E07 | ctg387 |
| bnlg1834 | b0039P10 |  |
| bnlg1834 | b0191G22 | ctg335 |
| bnlg1834 | b0049H01 | ctg335 |
| bnlg1834 | b0269C13 |  |
| bnlg1863 | b0013B10 | ctg340 |
| bnlg1863 | b0022H12 | ctg340 |
| bnlg1863 | b0144H19 | ctg340 |
| bnlg1863 | b0169G16 | ctg340 |
| bnlg1863 | b0216F07 |  |
| bnlg1866 | b0083L01 | ctg012 |
| bnlg1866 | b0131E08 | ctg012 |
| bnlg1866 | b0137D11 | ctg012 |
| bnlg1866 | b0057J15 | ctg012 |
| bnlg1866 | b0158P15 | ctg012 |
| bnlg1866 | b0210O13 | ctg012 |
| bnlg1867 | b0118B03 | ctg262 |
| bnlg1867 | b0188C21 | ctg262 |
| bnlg1867 | b0243D16 | ctg262 |
| bnlg1867 | b0282N16 | ctg262 |
| bnlg1879 | b0169I17 | ctg209 |
| bnlg1879 | b0103G12 | ctg209 |
| bnlg1879 | b0165N02 | ctg074 |
| bnlg1879 | b0189J04 | ctg209 |
| bnlg1879 | b0189N02 |  |
| bnlg1879 | b0080E11 |  |
| bnlg1892 | b0188G17 | ctg232 |
| bnlg1892 | b0188G17 | ctg232 |
| bnlg1892 | b0061D17 | ctg232 |
| bnlg1892 | b0092N05 | ctg232 |
| bnlg1892 | b0061D17 | ctg232 |
| bnlg1892 | b0061D17 | ctg232 |
| bnlg1892 | b0198L02 | ctg232 |
| bnlg1892 | b0092N05 | ctg232 |
| bnlg1892 | b0092N05 | ctg232 |
| bnlg1892 | b0198L02 | ctg232 |
| bnlg1892 | b0188G17 | ctg232 |
| bnlg1892 | b0198L02 | ctg232 |
| bnlg1892 | b0215M19 | ctg232 |
| bnlg1892 | b0256H11 |  |
| bnlg1892 | b0256H11 |  |
| bnlg1892 | b0256H11 |  |
| bnlg1892 | b0215M19 | ctg232 |
| bnlg1892 | b0215M19 | ctg232 |
| bnlg1893 | b0185J08 | ctg109 |
| bnlg1893 | b0030O09 | ctg109 |
| bnlg1893 | b0055G11 |  |
| bnlg1893 | b0272N09 | ctg109 |
| bnlg1902 | b0155L23 |  |
| bnlg1902 | b0005A18 |  |
| bnlg1902 | b0206K09 | ctg225 |
| bnlg1902 | b0237B24 | ctg225 |
| bnlg1902 | b0251B02 | ctg225 |
| bnlg1927 | b0131H08 | ctg057 |
| bnlg1927 | b0178P16 | ctg184 |
| bnlg1927 | b0128M21 |  |
| bnlg1927 | b0220G12 | ctg184 |
| bnlg1927 | b0263H08 | ctg184 |
| bnlg1940 | b0198B03 | ctg105 |
| bnlg1940 | b0079N24 | ctg105 |
| bnlg1940 | b0059J21 | ctg105 |
| bnlg1940 | b0271A09 |  |
| bnlg1940 | b0271G09 | ctg105 |
| bnlg1940 | b0278L11 |  |
| bnlg2046 | b0115F07 | ctg350 |
| bnlg2046 | b0175K24 | ctg350 |
| bnlg2046 | b0085D16 | ctg350 |
| bnlg2046 | b0171C07 | ctg350 |
| bnlg2046 | b0199L04 | ctg350 |
| bnlg2046 | b0135D16 | ctg350 |
| bnlg2046 | b0072K16 |  |
| bnlg2046 | b0141C07 | ctg419 |
| bnlg2057A | b0108E04 | ctg038 |
| bnlg2057B | b0050H06 |  |
| bnlg2077 | b0015N21 | ctg103 |
| bnlg2077 | b0046K15 | ctg120 |
| bnlg2077 | b0273P01 |  |
| bnlg2077 | b0267L01 |  |
| bnlg2082 | b0099F09 |  |
| bnlg2082 | b0166P12 | ctg188 |
| bnlg2082 | b0115F16 | ctg331 |
| bnlg2082 | b0096L01 | ctg331 |
| bnlg2082 | b0206K11 | ctg331 |
| bnlg2082 | b0212A04 | ctg031 |
| bnlg2086 | b0166D07 | ctg021 |
| bnlg2086 | b0064C01 |  |
| bnlg2086 | b0136I03 | ctg021 |
| bnlg2086 | b0089B07 | ctg021 |
| bnlg2086 | b0026C18 |  |
| bnlg2086 | b0132E09 | ctg021 |
| bnlg2086 | b0286O24 |  |
| bnlg2086 | b0212P09 | ctg021 |
| bnlg2086 | b0282A08 |  |
| bnlg2122 | b0127N23 | ctg296 |
| bnlg2122 | b0011P14 |  |
| bnlg2122 | b0055P18 | ctg368 |
| bnlg2122 | b0058L23 | ctg368 |
| bnlg2122 | b0274J01 |  |
| bnlg2122 | b0258G05 |  |
| bnlg2122 | b0219L08 | ctg387 |
| bnlg2122 | b0254F06 | ctg368 |
| bnlg2132 | b0277B17 | ctg293 |
| bnlg2162 | b0198F13 | ctg184 |
| bnlg2162 | b0007D24 | ctg184 |
| bnlg2162 | b0132F13 |  |
| bnlg2162 | b0104C04 |  |
| bnlg2162 | b0068N23 | ctg086 |
| bnlg2162 | b0109N04 |  |
| bnlg2162 | b0221L04 | ctg184 |
| bnlg2162 | b0212E05 |  |
| bnlg2181 | b0180O08 | ctg354 |
| bnlg2203 | b0274C19 | ctg306 |
| bnlg2203 | b0274C19 | ctg306 |
| bnlg2228 | b0033E20 |  |
| bnlg2228 | b0187A19 |  |
| bnlg2228 | b0123E19 | ctg047 |
| bnlg2228 | b0178A07 | ctg047 |
| bnlg2228 | b0272K01 | ctg047 |
| bnlg2228 | b0251C20 | ctg047 |
| bnlg2235 | b0182F16 | ctg086 |
| bnlg2235 | b0074H04 |  |
| bnlg2235 | b0099D17 |  |
| bnlg2235 | b0139M18 | ctg326 |
| bnlg2235 | b0093H07 |  |
| bnlg2235 | b0186K16 |  |
| bnlg2235 | b0240H03 | ctg326 |
| bnlg2235 | b0201F24 | ctg326 |
| bnlg2244 | b0136D20 |  |
| bnlg2244 | b0098K10 | ctg184 |
| bnlg2244 | b0133P16 | ctg184 |
| bnlg2244 | b0236C19 | ctg118 |
| bnlg2244 | b0250E01 |  |
| bnlg2244 | b0236C09 | ctg184 |
| bnlg2244 | b0277E16 | ctg184 |
| bnlg2249 | b0017G16 |  |
| bnlg2249 | b0119J23 | ctg283 |
| bnlg2259 | b0118L20 | ctg325 |
| bnlg2259 | b0123A05 | ctg325 |
| bnlg2259 | b0285L23 | ctg325 |
| bnlg2271 | b0062N07 | ctg321 |
| bnlg2271 | b0086M18 | ctg321 |
| bnlg2271 | b0082E23 |  |
| bnlg2271 | b0061N18 | ctg260 |
| bnlg2271 | b0119M03 |  |
| bnlg2271 | b0073C16 | ctg321 |
| bnlg2271 | b0271P18 |  |
| bnlg2277 | b0037F18 | ctg098 |
| bnlg2277 | b0149L11 | ctg071 |
| bnlg2277 | b0086C01 | ctg271 |
| bnlg2277 | b0005P06 |  |
| bnlg2277 | b0067F08 |  |
| bnlg2277 | b0133D12 |  |
| bnlg2277 | b0266P15 |  |
| bnlg2291 | b0001A23 | ctg182 |
| bnlg2291 | b0039H11 | ctg182 |
| bnlg2295 | b0089F23 | ctg020 |
| umc1006 | b0034B05 |  |
| umc1006 | b0100H15 |  |
| umc1006 | b0172G16 | ctg138 |
| umc1006 | b0172P05 | ctg271 |
| umc1006 | b0256G02 | ctg296 |
| umc1006 | b0283O21 |  |
| umc1006 | b0214N16 | ctg271 |
| umc1006 | b0285B08 | ctg029 |
| umc1006 | b0256L02 | ctg271 |
| umc1020 | b0019L11 | ctg008 |
| umc1020 | b0065P05 |  |
| umc1020 | b0017P05 | ctg373 |
| umc1020 | b0017P05 | ctg373 |
| umc1020 | b0090G13 |  |
| umc1024 | b0020M08 | ctg075 |
| umc1024 | b0268I19 | ctg075 |
| umc1029 | b0158M14 | ctg323 |
| umc1029 | b0140O18 | ctg323 |
| umc1029 | b0061I03 | ctg390 |
| umc1029 | b0061B03 | ctg323 |
| umc1029 | b0020I06 |  |
| umc1029 | b0139O18 | ctg318 |
| umc1029 | b0192A24 | ctg086 |
| umc1029 | b0252A24 | ctg323 |
| umc1030 | b0092E20 |  |
| umc1030 | b0273I02 | ctg114 |
| umc1031 | b0137F07 | ctg166 |
| umc1031 | b0033O16 |  |
| umc1031 | b0140B10 | ctg166 |
| umc1031 | b0082I03 | ctg166 |
| umc1031 | b0100D07 |  |
| umc1031 | b0059L20 | ctg166 |
| umc1031 | b0024O12 | ctg166 |
| umc1031 | b0286G16 | ctg166 |
| umc1035 | b0011K18 |  |
| umc1035 | b0009J11 | ctg041 |
| umc1035 | b0142D19 | ctg041 |
| umc1035 | b0011K11 |  |
| umc1035 | b0058B15 | ctg041 |
| umc1035 | b0187C19 | ctg417 |
| umc1035 | b0119J20 | ctg041 |
| umc1058 | b0130G03 | ctg202 |
| umc1058 | b0169L19 | ctg202 |
| umc1058 | b0028I11 | ctg202 |
| umc1058 | b0165I12 | ctg202 |
| umc1058 | b0183O16 | ctg202 |
| umc1058 | b0271I02 | ctg202 |
| umc1071 | b0096I24 |  |
| umc1071 | b0041C11 | ctg004 |
| umc1071 | b0176L24 |  |
| umc1071 | b0088L24 |  |
| umc1071 | b0205C12 |  |
| umc1076 | b0046H15 |  |
| umc1076 | b0007F20 | ctg030 |
| umc1076 | b0200N07 | ctg030 |
| umc1076 | b0247O17 | ctg030 |
| umc1076 | b0215O19 |  |
| umc1082 | b0178A11 | ctg057 |
| umc1082 | b0056P12 |  |
| umc1088 | b0070N04 |  |
| umc1088 | b0153N22 | ctg164 |
| umc1088 | b0282K02 | ctg164 |
| umc1088 | b0215D14 | ctg164 |
| umc1118 | b0128G06 | ctg064 |
| umc1118 | b0225G02 | ctg064 |
| umc1118 | b0211M08 | ctg064 |
| umc1123 | b0112H16 | ctg038 |
| umc1123 | b0112G20 | ctg038 |
| umc1123 | b0086P02 |  |
| umc1123 | b0148F20 | ctg086 |
| umc1123 | b0104J22 |  |
| umc1123 | b0006H02 | ctg038 |
| umc1123 | b0148F10 | ctg028 |
| umc1123 | b0064D23 | ctg038 |
| umc1123 | b0281K24 | ctg433 |
| umc1123 | b0277P24 |  |
| umc1129 | b0145C13 |  |
| umc1129 | b0108E24 | ctg063 |
| umc1133 | b0154J04 | ctg269 |
| umc1133 | b0126A17 | ctg269 |
| umc1133 | b0154J04 | ctg269 |
| umc1133 | b0257N18 | ctg269 |
| umc1133 | b0257N18 | ctg269 |
| umc1143 | b0005A07 |  |
| umc1143 | b0126A21 | ctg257 |
| umc1143 | b0076M24 |  |
| umc1143 | b0047F02 | ctg257 |
| umc1143 | b0084N09 | ctg257 |
| umc1143 | b0060A21 | ctg179 |
| umc1143 | b0205H01 |  |
| umc1147 | b0007D06 | ctg046 |
| umc1147 | b0098M10 |  |
| umc1147 | b0025L22 |  |
| umc1147 | b0131P01 | ctg046 |
| umc1147 | b0079F09 |  |
| umc1147 | b0217K22 | ctg046 |
| umc1147 | b0213A10 | ctg046 |
| umc1149 | b0076I04 | ctg325 |
| umc1149 | b0004I24 | ctg361 |
| umc1149 | b0075D24 | ctg361 |
| umc1149 | b0186E01 | ctg388 |
| umc1149 | b0243J15 |  |
| umc1149 | b0258K04 | ctg361 |
| umc1149 | b0232J03 | ctg361 |
| umc1149 | b0236D09 |  |
| umc1159 | b0038L18 |  |
| umc1159 | b0052C09 |  |
| umc1159 | b0205M14 |  |
| umc1159 | b0285G15 | ctg296 |
| umc1160 | b0099P15 |  |
| umc1160 | b0182P21 |  |
| umc1160 | b0182P21 |  |
| umc1166 | b0192I21 | ctg006 |
| umc1166 | b0066H12 |  |
| umc1166 | b0116D22 | ctg006 |
| umc1166 | b0080O01 | ctg006 |
| umc1166 | b0116K05 | ctg006 |
| umc1166 | b0055P12 | ctg006 |
| umc1166 | b0044J06 | ctg006 |
| umc1166 | b0056O01 |  |
| umc1166 | b0212O01 |  |
| umc1166 | b0257G03 | ctg006 |
| umc1176 | b0256C01 |  |
| umc1185 | b0181C02 |  |
| umc1185 | b0195A19 | ctg074 |
| umc1185 | b0242M19 | ctg074 |
| umc1191 | b0157M19 |  |
| umc1191 | b0036M14 |  |
| umc1191 | b0026I15 |  |
| umc1191 | b0156M14 | ctg376 |
| umc1191 | b0035B15 |  |
| umc1191 | b0156N06 | ctg074 |
| umc1191 | b0080J18 | ctg376 |
| umc1191 | b0282I08 | ctg376 |
| umc1191 | b0274G05 | ctg376 |
| umc1196 | b0076M13 | ctg417 |
| umc1196 | b0054G07 | ctg325 |
| umc1196 | b0145L04 | ctg417 |
| umc1196 | b0075H16 | ctg260 |
| umc1196 | b0077N24 |  |
| umc1196 | b0153F02 | ctg417 |
| umc1196 | b0252G09 | ctg417 |
| umc1196 | b0264I07 |  |
| umc1196 | b0208H13 | ctg304 |
| umc1196 | b0259E14 | ctg417 |
| umc1225 | b0068D01 | ctg267 |
| umc1225 | b0083I10 | ctg254 |
| umc1231 | b0007F12 | ctg386 |
| umc1245 | b0126F01 | ctg235 |
| umc1245 | b0114I09 | ctg046 |
| umc1245 | b0126B04 | ctg046 |
| umc1245 | b0005N22 |  |
| umc1245 | b0191O12 | ctg046 |
| umc1245 | b0132F01 | ctg046 |
| umc1245 | b0278M23 | ctg046 |
| umc1245 | b0202E07 |  |
| umc1245 | b0265B05 | ctg046 |
| umc1251 | b0159O10 | ctg323 |
| umc1251 | b0002N23 |  |
| umc1251 | b0159P10 |  |
| umc1251 | b0114C16 |  |
| umc1251 | b0006C16 | ctg246 |
| umc1251 | b0145L08 | ctg323 |
| umc1251 | b0006C23 |  |
| umc1251 | b0081F19 |  |
| umc1251 | b0288E18 | ctg323 |
| umc1251 | b0283E13 | ctg323 |
| umc1265 | b0177A12 |  |
| umc1265 | b0097C01 |  |
| umc1265 | b0283H19 | ctg069 |
| umc1268 | b0047A09 |  |
| umc1268 | b0113O21 | ctg363 |
| umc1268 | b0134C18 | ctg363 |
| umc1268 | b0267O09 | ctg363 |
| umc1272 | b0063H16 |  |
| umc1272 | b0239A17 | ctg413 |
| umc1301 | b0124N22 |  |
| umc1301 | b0199I15 |  |
| umc1301 | b0206K07 | ctg322 |
| umc1301 | b0266M04 |  |
| umc1304 | b0107L19 | ctg326 |
| umc1304 | b0051J19 |  |
| umc1304 | b0013L20 |  |
| umc1315 | b0114G23 | ctg218 |
| umc1315 | b0107B10 |  |
| umc1315 | b0072G24 |  |
| umc1315 | b0195O06 | ctg218 |
| umc1321 | b0079G09 | ctg034 |
| umc1321 | b0182H09 | ctg143 |
| umc1321 | b0083M21 | ctg270 |
| umc1321 | b0080C18 | ctg034 |
| umc1321 | b0186D09 | ctg358 |
| umc1321 | b0210M12 |  |
| umc1321 | b0268O13 | ctg034 |
| umc1321 | b0260H01 | ctg034 |
| umc1336 | b0046H07 |  |
| umc1336 | b0076K07 |  |
| umc1336 | b0232K07 | ctg406 |
| umc1337 | b0057B13 |  |
| umc1337 | b0204A10 | ctg393 |
| umc1349 | b0152C08 | ctg238 |
| umc1349 | b0165I23 |  |
| umc1349 | b0095H23 | ctg238 |
| umc1349 | b0032J16 | ctg238 |
| umc1349 | b0174N05 | ctg238 |
| umc1358 | b0054F14 | ctg043 |
| umc1358 | b0162C10 | ctg043 |
| umc1358 | b0172D15 |  |
| umc1358 | b0211N22 | ctg043 |
| umc1358 | b0257C05 | ctg043 |
| umc1363 | b0168M24 | ctg118 |
| umc1363 | b0257I20 | ctg004 |
| umc1388 | b0098M21 | ctg284 |
| umc1388 | b0198H06 | ctg284 |
| umc1388 | b0224K16 | ctg284 |
| umc1388 | b0258L02 | ctg284 |
| umc1392 | b0001G03 | ctg115 |
| umc1392 | b0001G03 | ctg115 |
| umc1396 | b0123D02 |  |
| umc1396 | b0043M12 |  |
| umc1396 | b0239F06 | ctg039 |
| umc1396 | b0284N18 | ctg039 |
| umc1397 | b0174J04 | ctg011 |
| umc1397 | b0126N09 | ctg011 |
| umc1397 | b0183O03 | ctg011 |
| umc1397 | b0275J03 | ctg011 |
| umc1403 | b0021G07 | ctg011 |
| umc1403 | b0179C10 | ctg011 |
| umc1403 | b0089I23 | ctg349 |
| umc1403 | b0102H05 | ctg011 |
| umc1403 | b0166H06 | ctg011 |
| umc1403 | b0261B23 | ctg011 |
| umc1403 | b0263F04 | ctg011 |
| umc1406 | b0038J06 |  |
| umc1406 | b0075H02 | ctg325 |
| umc1406 | b0167L21 |  |
| umc1406 | b0259M03 | ctg325 |
| umc1406 | b0221M18 |  |
| umc1412 | b0033L15 | ctg325 |
| umc1412 | b0176H01 | ctg141 |
| umc1412 | b0193A21 | ctg453 |
| umc1412 | b0248O19 | ctg325 |
| umc1412 | b0250M02 | ctg014 |
| umc1412 | b0243L05 | ctg104 |
| umc1421 | b0082E09 |  |
| umc1422 | b0072O04 |  |
| umc1422 | b0019H22 | ctg071 |
| umc1422 | b0001F06 |  |
| umc1422 | b0212C11 | ctg071 |
| umc1423 | b0097M12 | ctg204 |
| umc1423 | b0014O22 | ctg204 |
| umc1423 | b0085D12 | ctg416 |
| umc1423 | b0004G02 | ctg445 |
| umc1423 | b0115D03 | ctg204 |
| umc1423 | b0087G12 | ctg204 |
| umc1423 | b0205A14 | ctg204 |
| umc1423 | b0226J11 | ctg204 |
| umc1423 | b0270F02 | ctg204 |
| umc1447 | b0016D13 |  |
| umc1447 | b0080D03 | ctg217 |
| umc1447 | b0086N21 | ctg217 |
| umc1447 | b0055G07 |  |
| umc1449 | b0041D20 | ctg121 |
| umc1449 | b0155O01 | ctg121 |
| umc1449 | b0244J10 | ctg121 |
| umc1479 | b0055D11 | ctg011 |
| umc1479 | b0119D04 | ctg011 |
| umc1479 | b0057C21 |  |
| umc1479 | b0161N23 |  |
| umc1479 | b0058C15 |  |
| umc1479 | b0039E23 |  |
| umc1479 | b0107E19 | ctg011 |
| umc1479 | b0039C23 |  |
| umc1479 | b0165D21 | ctg011 |
| umc1493 | b0039L17 |  |
| umc1501 | b0031D19 |  |
| umc1501 | b0253E04 |  |
| umc1505 | b0113M05 |  |
| umc1505 | b0104L20 |  |
| umc1505 | b0037O06 | ctg391 |
| umc1506 | b0273M08 | ctg414 |
| umc1515 | b0116M15 | ctg026 |
| umc1515 | b0009D04 |  |
| umc1515 | b0270P03 |  |
| umc1515 | b0259G22 | ctg026 |
| umc1523a | b0060M10 | ctg204 |
| umc1523a | b0067K19 | ctg204 |
| umc1523b | b0084H12 | ctg057 |
| umc1524 | b0037O13 | ctg249 |
| umc1524 | b0088J02 |  |
| umc1524 | b0128M16 | ctg249 |
| umc1553 | b0174G05 | ctg062 |
| umc1553 | b0100C02 | ctg062 |
| umc1553 | b0099J04 | ctg062 |
| umc1553 | b0167A24 | ctg062 |
| umc1553 | b0229A03 | ctg062 |
| umc1553 | b0272G06 |  |
| umc1566 | b0103O04 | ctg002 |
| umc1566 | b0038L18 |  |
| umc1566 | b0042O18 | ctg413 |
| umc1566 | b0119O02 | ctg002 |
| umc1566 | b0085P08 | ctg002 |
| umc1566 | b0027F19 |  |
| umc1566 | b0030O18 |  |
| umc1566 | b0242K13 | ctg002 |
| umc1580 | b0185D02 | ctg078 |
| umc1580 | b0093F22 | ctg230 |
| umc1580 | b0157O09 |  |
| umc1580 | b0072D13 |  |
| umc1580 | b0072F13 |  |
| umc1580 | b0112O08 |  |
| umc1580 | b0242M04 | ctg078 |
| umc1586 | b0099A02 | ctg373 |
| umc1586 | b0191C18 | ctg373 |
| umc1587 | b0020O17 | ctg111 |
| umc1587 | b0041L10 | ctg208 |
| umc1587 | b0155I23 |  |
| umc1587 | b0026O17 |  |
| umc1587 | b0057O21 |  |
| umc1587 | b0074A11 | ctg208 |
| umc1591 | b0122G23 |  |
| umc1591 | b0090P05 |  |
| umc1591 | b0101I12 | ctg227 |
| umc1591 | b0253A12 |  |
| umc1591 | b0279M24 | ctg227 |
| umc1595 | b0070J23 |  |
| umc1595 | b0122O07 | ctg272 |
| umc1595 | b0096G03 |  |
| umc1596 | b0117C18 | ctg141 |
| umc1596 | b0118K11 | ctg371 |
| umc1601 | b0169A24 |  |
| umc1601 | b0055B13 |  |
| umc1604 | b0134K01 | ctg162 |
| umc1604 | b0030E18 | ctg104 |
| umc1604 | b0047H20 |  |
| umc1604 | b0135H16 | ctg104 |
| umc1604 | b0031I15 |  |
| umc1604 | b0095G07 | ctg104 |
| umc1604 | b0136H01 |  |
| umc1604 | b0218N07 | ctg104 |
| umc1604 | b0246G19 |  |
| umc1607 | b0029I16 |  |
| umc1607 | b0013P07 | ctg363 |
| umc1607 | b0020C05 | ctg363 |
| umc1607 | b0264G22 |  |
| umc1630 | b0077L07 |  |
| umc1630 | b0196N20 | ctg430 |
| umc1630 | b0038C20 | ctg064 |
| umc1630 | b0216A17 | ctg064 |
| umc1630 | b0258C14 | ctg064 |
| umc1630 | b0260B10 | ctg064 |
| umc1632 | b0103P02 | ctg296 |
| umc1632 | b0145K23 | ctg296 |
| umc1632 | b0136O14 | ctg296 |
| umc1632 | b0015O21 |  |
| umc1632 | b0013D21 |  |
| umc1632 | b0203I20 | ctg296 |
| umc1632 | b0206H09 | ctg296 |
| umc1632 | b0202O14 |  |
| umc1636 | b0110C20 | ctg373 |
| umc1636 | b0044F06 | ctg373 |
| umc1636 | b0053F12 |  |
| umc1636 | b0166F03 | ctg373 |
| umc1636 | b0231M13 | ctg373 |
| umc1636 | b0211E02 | ctg373 |
| umc1636 | b0275G22 |  |
| umc1638 | b0086G23 | ctg067 |
| umc1638 | b0098O03 | ctg366 |
| umc1638 | b0171O04 | ctg366 |
| umc1638 | b0137G15 | ctg092 |
| umc1638 | b0016C10 |  |
| umc1638 | b0138L05 |  |
| umc1638 | b0007G22 |  |
| umc1638 | b0085L09 |  |
| umc1638 | b0202M16 |  |
| umc1638 | b0232I23 |  |
| umc1641 | b0080O17 | ctg151 |
| umc1641 | b0169E11 | ctg151 |
| umc1641 | b0030K03 | ctg151 |
| umc1641 | b0197K02 |  |
| umc1644 | b0031D24 |  |
| umc1644 | b0077H20 |  |
| umc1644 | b0095D15 |  |
| umc1655 | b0023L18 | ctg116 |
| umc1655 | b0099P08 | ctg116 |
| umc1655 | b0192M22 | ctg116 |
| umc1655 | b0100O05 | ctg132 |
| umc1655 | b0271G07 | ctg116 |
| umc1655 | b0241B09 | ctg116 |
| umc1655 | b0268H23 |  |
| umc1658 | b0024D03 | ctg091 |
| umc1658 | b0143J04 | ctg091 |
| umc1658 | b0065E20 | ctg091 |
| umc1658 | b0100C23 | ctg091 |
| umc1658 | b0179F09 | ctg091 |
| umc1658 | b0156N11 | ctg091 |
| umc1658 | b0226L04 | ctg091 |
| umc1658 | b0276H08 | ctg091 |
| umc1658 | b0279E20 |  |
| umc1672 | b0066L24 |  |
| umc1672 | b0196I01 | ctg182 |
| umc1672 | b0015I10 |  |
| umc1672 | b0283N10 | ctg293 |
| umc1672 | b0284J01 | ctg293 |
| umc1672 | b0266P13 |  |
| umc1672 | b0267M21 |  |
| umc1676 | b0151O04 | ctg031 |
| umc1676 | b0123E03 |  |
| umc1676 | b0145B14 | ctg031 |
| umc1676 | b0212A04 | ctg031 |
| umc1676 | b0240N11 | ctg031 |
| umc1680 | b0094I21 |  |
| umc1680 | b0112K15 |  |
| umc1680 | b0271M20 | ctg250 |
| umc1685 | b0197H01 | ctg005 |
| umc1685 | b0044J20 | ctg005 |
| umc1685 | b0088L14 |  |
| umc1685 | b0008N14 | ctg005 |
| umc1685 | b0093P03 |  |
| umc1688 | b0122G17 | ctg376 |
| umc1688 | b0054C01 | ctg183 |
| umc1688 | b0103H10 | ctg376 |
| umc1688 | b0059M12 | ctg376 |
| umc1688 | b0210P09 |  |
| umc1688 | b0239P18 |  |
| umc1703 | b0070G04 |  |
| umc1703 | b0181P16 |  |
| umc1707 | b0087F19 | ctg203 |
| umc1707 | b0124B24 | ctg203 |
| umc1707 | b0132B21 |  |
| umc1707 | b0080H21 | ctg444 |
| umc1707 | b0105C02 | ctg203 |
| umc1707 | b0132G14 | ctg223 |
| umc1707 | b0275G14 | ctg203 |
| umc1707 | b0269D14 | ctg203 |
| umc1713 | b0179O12 | ctg318 |
| umc1713 | b0069C12 |  |
| umc1713 | b0130F07 | ctg318 |
| umc1713 | b0269A02 |  |
| umc1713 | b0246M02 |  |
| umc1713 | b0211M06 | ctg318 |
| umc1717 | b0103I20 |  |
| umc1717 | b0045O18 | ctg117 |
| umc1717 | b0079I11 |  |
| umc1717 | b0037P06 |  |
| umc1717 | b0025P18 |  |
| umc1717 | b0073J12 | ctg179 |
| umc1717 | b0094P22 | ctg149 |
| umc1717 | b0021A12 | ctg343 |
| umc1717 | b0248O22 | ctg074 |
| umc1733 | b0095M11 | ctg389 |
| umc1733 | b0126C18 |  |
| umc1733 | b0021O03 | ctg389 |
| umc1733 | b0243D13 | ctg389 |
| umc1733 | b0225M19 |  |
| umc1744 | b0107B16 |  |
| umc1744 | b0037A02 | ctg064 |
| umc1744 | b0275F19 | ctg064 |
| umc1744 | b0239K20 |  |
| umc1757 | b0184G10 | ctg156 |
| umc1757 | b0085H14 | ctg156 |
| umc1757 | b0241C03 |  |
| umc1757 | b0271L22 | ctg156 |
| umc1759 | b0184G10 | ctg156 |
| umc1759 | b0236H06 | ctg156 |
| umc1776 | b0099C24 | ctg074 |
| umc1776 | b0167A09 | ctg074 |
| umc1776 | b0160F13 | ctg074 |
| umc1776 | b0092E12 | ctg074 |
| umc1776 | b0056G05 | ctg074 |
| umc1776 | b0286I09 | ctg074 |
| umc1776 | b0272B06 |  |
| umc1796 | b0218P06 |  |
| umc1808 | b0158K02 |  |
| umc1819 | b0105B08 | ctg066 |
| umc1819 | b0107A21 |  |
| umc1819 | b0035C23 | ctg066 |
| umc1819 | b0097P13 | ctg066 |
| umc1819 | b0206E18 | ctg066 |
| umc1825 | b0085I08 |  |
| umc1825 | b0145A10 | ctg044 |
| umc1825 | b0192L04 | ctg144 |
| umc1825 | b0142D17 | ctg144 |
| umc1825 | b0091L07 | ctg182 |
| umc1825 | b0254P13 |  |
| umc1825 | b0254A04 | ctg138 |
| umc1825 | b0253L05 | ctg261 |
| umc1825 | b0253L07 |  |
| umc1825 | b0240H22 | ctg144 |
| umc1838 | b0067P04 |  |
| umc1838 | b0099D21 |  |
| umc1838 | b0133P06 | ctg344 |
| umc1838 | b0259H08 | ctg049 |
| umc1838 | b0211P04 | ctg091 |
| umc1838 | b0213D21 | ctg049 |
| umc1857 | b0094G20 | ctg277 |
| umc1857 | b0004H10 |  |
| umc1857 | b0030O11 |  |
| umc1857 | b0236N16 | ctg277 |
| umc1857 | b0223J03 | ctg277 |
| umc1857 | b0209I06 | ctg277 |
| umc1857 | b0214D02 |  |
| umc1875 | b0111M22 | ctg217 |
| umc1875 | b0155D24 |  |
| umc1875 | b0087F01 |  |
| umc1875 | b0263L05 | ctg199 |
| umc1875 | b0211A12 |  |
| umc1880 | b0137D11 | ctg012 |
| umc1880 | b0158P15 | ctg012 |
| umc1880 | b0083L01 | ctg012 |
| umc1880 | b0057J15 | ctg012 |
| umc1880 | b0131E08 | ctg012 |
| umc1880 | b0210O13 | ctg012 |
| umc1888 | b0138O14 | ctg322 |
| umc1888 | b0004L23 | ctg375 |
| umc1888 | b0187G22 | ctg322 |
| umc1888 | b0023I04 |  |
| umc1888 | b0092B18 |  |
| umc1888 | b0003M20 | ctg322 |
| umc1888 | b0235A15 | ctg322 |
| umc1892 | b0102A24 | ctg111 |
| umc1892 | b0018O22 | ctg111 |
| umc1892 | b0142O03 | ctg111 |
| umc1892 | b0002B19 |  |
| umc1899 | b0145N14 | ctg184 |
| umc1899 | b0117G01 | ctg184 |
| umc1899 | b0165L14 | ctg068 |
| umc1899 | b0166L05 | ctg184 |
| umc1899 | b0009L14 |  |
| umc1903 | b0140C23 | ctg033 |
| umc1903 | b0068F11 | ctg091 |
| umc1903 | b0171F01 | ctg033 |
| umc1903 | b0179A17 | ctg451 |
| umc1903 | b0180L21 | ctg033 |
| umc1903 | b0126A08 | ctg033 |
| umc1903 | b0278F06 | ctg033 |
| umc1903 | b0264H18 | ctg033 |
| umc1903 | b0268D06 | ctg171 |
| umc1906 | b0084A19 | ctg033 |
| umc1906 | b0284F02 | ctg033 |
| umc1906 | b0241P13 | ctg033 |
| umc1911 | b0146I18 |  |
| umc1911 | b0103D04 | ctg412 |
| umc1911 | b0248N05 | ctg412 |
| umc1911 | b0214P17 | ctg412 |
| umc1912 | b0076I22 |  |
| umc1912 | b0092F18 | ctg087 |
| umc1912 | b0032F03 | ctg287 |
| umc1912 | b0082H20 | ctg287 |
| umc1912 | b0074F22 |  |
| umc1912 | b0273D03 | ctg287 |
| umc1912 | b0235A16 | ctg269 |
| umc1913 | b0117K16 | ctg328 |
| umc1913 | b0196O11 | ctg328 |
| umc1913 | b0081A15 |  |
| umc1913 | b0077N16 |  |
| umc1913 | b0065B19 |  |
| umc1913 | b0126E17 | ctg328 |
| umc1913 | b0244N09 |  |
| umc1913 | b0273P08 |  |
| umc1913 | b0226N10 | ctg328 |
| umc1914 | b0116C10 | ctg052 |
| umc1914 | b0157J06 |  |
| umc1914 | b0218L08 |  |
| umc1914 | b0221C09 |  |
| umc1917 | b0045O01 | ctg014 |
| umc1917 | b0154O10 | ctg014 |
| umc1917 | b0124F14 | ctg014 |
| umc1917 | b0240J01 | ctg014 |
| umc1917 | b0247E11 | ctg014 |
| umc1917 | b0237B20 | ctg014 |
| umc1917 | b0241O01 |  |
| umc1917 | b0256H24 | ctg014 |
| umc1918 | b0131E23 | ctg276 |
| umc1918 | b0126G18 | ctg276 |
| umc1918 | b0097C14 |  |
| umc1918 | b0173I21 | ctg276 |
| umc1918 | b0064B08 | ctg276 |
| umc1918 | b0001C14 |  |
| umc1918 | b0181A20 |  |
| umc1918 | b0002P14 | ctg276 |
| umc1919 | b0039I18 |  |
| umc1919 | b0052A18 | ctg162 |
| umc1919 | b0104H11 | ctg039 |
| umc1919 | b0215E08 | ctg039 |
| umc1919 | b0279A09 | ctg039 |
| umc1919 | b0211O10 | ctg039 |
| umc1923 | b0115B17 |  |
| umc1923 | b0016H04 | ctg092 |
| umc1924 | b0135C08 | ctg041 |
| umc1924 | b0138K06 | ctg041 |
| umc1924 | b0264J24 |  |
| umc1924 | b0275F10 |  |
| umc1924 | b0249F02 | ctg041 |
| umc1924 | b0245L15 |  |
| umc1924 | b0250H08 | ctg041 |
| umc1924 | b0264I24 |  |
| umc1925 | b0142F01 | ctg041 |
| umc1925 | b0142F01 | ctg041 |
| umc1925 | b0075P03 | ctg219 |
| umc1925 | b0117J08 | ctg041 |
| umc1925 | b0023H09 | ctg041 |
| umc1925 | b0023H09 | ctg041 |
| umc1925 | b0170F05 |  |
| umc1925 | b0219L03 | ctg041 |
| umc1929 | b0039M15 |  |
| umc1929 | b0147P16 | ctg310 |
| umc1932 | b0055O18 | ctg308 |
| umc1932 | b0097E23 |  |
| umc1932 | b0276K06 | ctg308 |
| umc1936 | b0107C21 | ctg322 |
| umc1936 | b0121J09 | ctg322 |
| umc1936 | b0148J17 |  |
| umc1936 | b0258A07 |  |
| umc1943 | b0131G19 | ctg156 |
| umc1943 | b0263J23 | ctg156 |
| umc1955 | b0070E13 |  |
| umc1955 | b0038M08 |  |
| umc1957 | b0025N23 |  |
| umc1957 | b0199N23 |  |
| umc1957 | b0200H12 | ctg368 |
| umc1959 | b0026D24 | ctg354 |
| umc1959 | b0073B17 |  |
| umc1959 | b0145B01 | ctg354 |
| umc1959 | b0184J18 | ctg354 |
| umc1959 | b0075A09 | ctg354 |
| umc1959 | b0049A08 | ctg354 |
| umc1959 | b0160B22 | ctg354 |
| umc1959 | b0197E20 | ctg354 |
| umc1959 | b0221F13 | ctg122 |
| umc1959 | b0217D13 | ctg354 |
| umc1962 | b0200A23 | ctg397 |
| umc1962 | b0050J18 |  |
| umc1962 | b0125P17 | ctg397 |
| umc1962 | b0177J18 | ctg397 |
| umc1962 | b0244G22 | ctg397 |
| umc1962 | b0225O08 | ctg397 |
| umc1969 | b0060D03 |  |
| umc1973 | b0072H05 | ctg132 |
| umc1973 | b0154G19 | ctg132 |
| umc1973 | b0095K23 | ctg108 |
| umc1973 | b0099F09 |  |
| umc1973 | b0072O18 |  |
| umc1973 | b0232B24 | ctg132 |
| umc1973 | b0251K23 |  |
| umc1978 | b0112C15 | ctg415 |
| umc1978 | b0111C09 | ctg298 |
| umc1978 | b0189D02 |  |
| umc1978 | b0013E07 | ctg298 |
| umc1978 | b0140F01 |  |
| umc1978 | b0230L15 |  |
| umc1978 | b0246E21 |  |
| umc1987a | b0110M20 | ctg318 |
| umc1987a | b0014H02 | ctg318 |
| umc1987a | b0036A04 |  |
| umc1987a | b0264J14 | ctg318 |
| umc1987a | b0260J24 | ctg318 |
| umc1987a | b0264P21 |  |
| umc1987b | b0160E23 | ctg098 |
| umc1987b | b0048J14 | ctg076 |
| umc1987b | b0023L02 | ctg098 |
| umc1987b | b0156J09 | ctg098 |
| umc1987b | b0194C16 | ctg098 |
| umc1987b | b0275P14 |  |
| umc1987b | b0268E23 |  |
| umc1987b | b0231K05 | ctg098 |
| umc1991 | b0017G05 | ctg432 |
| umc1991 | b0121I17 | ctg050 |
| umc1991 | b0114G05 | ctg050 |
| umc1991 | b0111A16 |  |
| umc1991 | b0036L22 | ctg050 |
| umc1991 | b0247H06 | ctg081 |
| umc1991 | b0209G05 | ctg411 |
| umc1999 | b0154N24 | ctg196 |
| umc1999 | b0147A16 | ctg196 |
| umc1999 | b0045D06 | ctg196 |
| umc2002 | b0085C10 | ctg123 |
| umc2002 | b0084E04 | ctg123 |
| umc2002 | b0269B16 | ctg123 |
| umc2002 | b0242K01 | ctg123 |
| umc2002 | b0229H23 |  |
| umc2006 | b0061O09 | ctg281 |
| umc2006 | b0043D18 |  |
| umc2006 | b0024L20 | ctg281 |
| umc2006 | b0246P19 |  |
| umc2006 | b0238F24 | ctg281 |
| umc2006 | b0230C11 | ctg247 |
| umc2006 | b0229C23 |  |
| umc2006 | b0229D23 | ctg172 |
| umc2012 | b0029F19 | ctg004 |
| umc2012 | b0112G01 | ctg004 |
| umc2012 | b0152B22 | ctg004 |
| umc2012 | b0200K20 | ctg004 |
| umc2012 | b0264D15 |  |
| umc2012 | b0246O15 |  |
| umc2020 | b0111D09 | ctg126 |
| umc2020 | b0160A05 | ctg091 |
| umc2020 | b0158A05 | ctg126 |
| umc2020 | b0160A20 | ctg126 |
| umc2020 | b0160A20 | ctg126 |
| umc2020 | b0090J13 | ctg118 |
| umc2020 | b0112D13 | ctg250 |
| umc2020 | b0241N15 | ctg040 |
| umc2020 | b0241N06 | ctg126 |
| umc2036 | b0032K12 | ctg206 |
| umc2036 | b0173P16 |  |
| umc2036 | b0025F05 | ctg206 |
| umc2036 | b0225M04 | ctg206 |
| umc2047 | b0197H18 | ctg055 |
| umc2047 | b0104D22 |  |
| umc2050 | b0034L23 |  |
| umc2050 | b0102N07 | ctg140 |
| umc2050 | b0095B12 |  |
| umc2050 | b0253M21 | ctg140 |
| umc2050 | b0239B02 | ctg140 |
| umc2059 | b0189N24 | ctg291 |
| umc2059 | b0122K11 | ctg291 |
| umc2059 | b0189I08 | ctg164 |
| umc2059 | b0077L12 |  |
| umc2059 | b0096G10 | ctg291 |
| umc2059 | b0075I01 | ctg291 |
| umc2059 | b0201K06 |  |
| umc2059 | b0221A23 |  |
| umc2059 | b0288J14 | ctg291 |
| umc2082 | b0011P17 |  |
| umc2082 | b0112I06 | ctg158 |
| umc2082 | b0131J06 | ctg158 |
| umc2082 | b0072I10 |  |
| umc2082 | b0220M06 |  |
| umc2082 | b0227M14 |  |
| umc2105 | b0125E24 | ctg111 |
| umc2105 | b0162O10 | ctg111 |
| umc2105 | b0197A10 | ctg111 |
| umc2105 | b0162E09 | ctg124 |
| umc2105 | b0186D06 | ctg111 |
| umc2105 | b0158D09 | ctg111 |
| umc2105 | b0049G20 | ctg111 |
| umc2105 | b0162O09 |  |
| umc2105 | b0221D11 | ctg111 |
| umc2105 | b0276K08 | ctg111 |
| umc2112 | b0139O10 | ctg021 |
| umc2112 | b0152D21 |  |
| umc2112 | b0139O06 | ctg354 |
| umc2112 | b0139G06 | ctg021 |
| umc2112 | b0190J18 | ctg021 |
| umc2112 | b0033O09 | ctg021 |
| umc2112 | b0225E13 | ctg021 |
| umc2112 | b0211K05 | ctg021 |
| umc2112 | b0216P12 | ctg037 |
| umc2112 | b0205C20 |  |
| umc2121 | b0002L09 |  |
| umc2121 | b0198J05 |  |
| umc2121 | b0119D22 | ctg385 |
| umc2124 | b0008P16 |  |
| umc2124 | b0085B05 | ctg108 |
| umc2124 | b0126L02 | ctg014 |
| umc2124 | b0146P03 |  |
| umc2124 | b0119G24 |  |
| umc2124 | b0279D05 |  |
| umc2124 | b0275K08 | ctg014 |
| umc2126 | b0086N24 | ctg269 |
| umc2126 | b0141N11 | ctg091 |
| umc2126 | b0085D04 | ctg220 |
| umc2126 | b0013N03 | ctg423 |
| umc2126 | b0143K03 | ctg260 |
| umc2126 | b0081J19 |  |
| umc2126 | b0008O12 |  |
| umc2126 | b0011K04 |  |
| umc2126 | b0169D04 | ctg064 |
| umc2126 | b0079C19 | ctg193 |
| umc2126 | b0218O17 | ctg138 |
| umc2126 | b0220N07 | ctg419 |
| umc2129 | b0105J09 | ctg098 |
| umc2129 | b0103F04 | ctg223 |
| umc2129 | b0039I07 | ctg098 |
| umc2129 | b0150O09 | ctg098 |
| umc2129 | b0116I19 | ctg098 |
| umc2129 | b0100O16 | ctg098 |
| umc2129 | b0044A01 |  |
| umc2129 | b0210B16 | ctg254 |
| umc2129 | b0253F18 |  |
| umc2139 | b0051P09 |  |
| umc2139 | b0174N09 | ctg195 |
| umc2149 | b0062A04 | ctg057 |
| umc2149 | b0057D12 | ctg057 |
| umc2149 | b0220G07 | ctg057 |
| umc2149 | b0204G03 | ctg057 |
| umc2178 | b0130I01 | ctg091 |
| umc2178 | b0137J17 | ctg162 |
| umc2178 | b0036G22 |  |
| umc2178 | b0099J02 | ctg091 |
| umc2178 | b0137O20 | ctg091 |
| umc2178 | b0211P04 | ctg091 |
| umc2178 | b0276F20 | ctg091 |
| umc2181 | b0198K10 | ctg048 |
| umc2181 | b0172M20 | ctg048 |
| umc2181 | b0007B20 | ctg048 |
| umc2181 | b0009B18 |  |
| umc2181 | b0091K14 | ctg048 |
| umc2181 | b0092H20 | ctg048 |
| umc2181 | b0257H14 | ctg048 |
| umc2187 | b0028B19 | ctg183 |
| umc2187 | b0162L10 |  |
| umc2187 | b0073K21 | ctg188 |
| umc2187 | b0193L15 |  |
| umc2187 | b0193H04 | ctg188 |
| umc2187 | b0207P05 | ctg188 |
| umc2187 | b0235G24 |  |
| umc2189 | b0015B17 | ctg385 |
| umc2189 | b0195B17 |  |
| umc2189 | b0110H07 | ctg411 |
| umc2189 | b0050J10 |  |
| umc2189 | b0074H07 | ctg179 |
| umc2189 | b0197G11 |  |
| umc2189 | b0276F14 | ctg057 |
| umc2189 | b0230H07 |  |
| umc2189 | b0229B24 |  |
| umc2189 | b0229B11 |  |
