# Supplementary material for: A BAC pooling strategy combined with PCR-based screenings in a large, highly repetitive genome enables integration of the maize genetic and physical maps
Source: BMC Genomics. 2007 Feb 9;8:47. doi: 10.1186/1471-2164-8-47 (PMC1821331; doi:10.1186/1471-2164-8-47)
Supplement: Additional File 1 — BAC pool DNA isolation [file 1471-2164-8-47-S1.doc]

Supporting information; included for PNAS web site display.

**METHOD**

**BAC pool DNA isolation**

Each pool contained approximately 100 ml of culture. Individually, pooled cultures were transferred to 250-ml centrifuge bottles and incubated on ice for 15 min. The cells were collected by centrifugation at 4400 g for 15 min at 4 °C. The supernatant was removed and the pellets drained. Cell pellets were respun briefly to pipette out the remaining TB media. The cell pellet was resuspended in 2.4 ml of solution I (50 mM Tris-HCl pH 7.5, 50 mM EDTA pH 7.5) by vortexing and pipetting the solution up and down. The resuspended cell pellet was transferred to a 50-ml Oakridge centrifuge tube and 4.8 ml of freshly prepared solution II (0.2 M NaOH, 1% SDS) was added. The sample was mixed by inversion 10-12 times and incubated 15 min at room temperature. Next, 3.6 ml of ice-cold solution III (3 M potassium acetate pH 5.2) was added and the sample was mixed by inversion 12 times. Following incubation in an ice-water bath for 20 min, the sample was centrifuged at 31,000 g for 20 min at 30 °C. The supernatant was transferred to a new tube and respun at 31,000 g for 20 min at 30 °C to completely clarify the supernatant. The supernatant was transferred into another clean Oakridge centrifuge tube containing 10 ml of isopropanol, mixed and incubated overnight at –20 °C. The precipitated nucleic acid was centrifuged at 13,800 g for 30 min at 4 °C, washed twice (70% ethanol first wash, 80% ethanol second wash), air-dried, and resuspended in TE buffer containing 20 μg/ml RNase A. The sample was incubated at 37 °C for 1 hr. 30 min to degrade RNA, transferred to a 1.5ml microcentrifuge tube and centrifuged at 15,000 g for 5 min to remove any residual insoluble material. The supernatant was transferred to a new tube and the BAC DNA quantified by fluorimetry. Average yields for BAC pool DNAs were approximately 1.5 μg BAC DNA/ml of starting culture.

### 
